# Synthetic Capillaries to Control Microscopic Blood Flow

**DOI:** 10.1038/srep21885

**Published:** 2016-02-24

**Authors:** K. Sarveswaran, V. Kurz, Z. Dong, T. Tanaka, S. Penny, G. Timp

**Affiliations:** 1 Depts. Biological Science and Electrical Engineering, 316 Stinson-Remick Hall, University of Notre Dame, Notre Dame, IN 46556.

## Abstract

Capillaries pervade human physiology. The mean intercapillary distance is only about 100 μm in human tissue, which indicates the extent of nutrient diffusion. In engineered tissue the lack of capillaries, along with the associated perfusion, is problematic because it leads to hypoxic stress and necrosis. However, a capillary is not easy to engineer due to its complex cytoarchitecture. Here, it is shown that it is possible to create *in vitro,* in about 30 min, a tubular microenvironment with an elastic modulus and porosity consistent with human tissue that functionally mimicks a *bona fide* capillary using “live cell lithography”(LCL) to control the type and position of cells on a composite hydrogel scaffold. Furthermore, it is established that these constructs support the forces associated with blood flow, and produce nutrient gradients similar to those measured *in vivo*. With LCL, capillaries can be constructed with single cell precision—no other method for tissue engineering offers such precision. Since the time required for assembly scales with the number of cells, this method is likely to be adapted first to create minimal functional units of human tissue that constitute organs, consisting of a heterogeneous population of 100–1000 cells, organized hierarchically to express a predictable function.

Tissue engineering is a cure. Eventually, tissue may be engineered for organ replacement and regenerative medicine, but *in vitro* models engineered to accurately emulate human physiology are more likely to have an effect on health sooner, for example, in drug discovery^1^. *In vitro* microfluidic organs-on-a-chip offer the promise to recapitulate the minimal functional units (MFU) found in major organs, such as the acinus (liver), nephron (kidney) or alveoli (lung), but so far have failed to reproduce the micro-scale hierarchical organization, spatial heterogeneity and cell-matrix interactions found in organoids or tissue sections^2^. Responding to these challenges, a number of ways have been developed to co-culture two or more cell types and control the cell positions^3^,^4^,^5^, which include rapid casting^5^ and bioprinting^6^,^7^, and each has advantages and limitations. The prior work has revealed several technical obstacles that still have to be overcome to create functional engineered tissue;^1^,^8^ among them are 1. engineered tissue lacks a hierarchical organization required to deliver specific biological functions; 2. the mechanics of the engineered tissue are weak compared to *in vivo* tissue; 3. a *bona fide* extracellular matrix (ECM) is cell-specific^9^, but the scaffolds used to engineer tissue are not; and 4. engineered tissue lacks the microcirculation and the complex vasculature required for perfusion to avoid necrosis—the volume of engineered tissue cannot exceed the diffusion limit^10^.

“Live cell lithography” (LCL) may possibly resolve these issues. LCL uses multiple optical tweezers to precisely position cells in three-dimensions (3D) in a photo-polymerized hydrogel scaffold—creating “living voxels” with exposed cell surfaces^11^,^12^. Furthermore, by stitching the living voxels together, a cytoarchitecture of any size, shape and constituency can be formed and embedded in a microfluidic device for testing. In these constructs, the hydrogel scaffold performs a function similar to the natural ECM, but it is synthetic, sterile and can be hybridized. Importantly, with LCL, it is possible to tailor the microenvironment of each cell-type by photo-polymerizing a specific pre-polymer in each voxel to form cell-specific scaffolds.

As a demonstration, by controlling the type and position of cells on a composite, cell-specific hydrogel scaffold, LCL was used to create perfusable synthetic capillaries in about 30 min. Interestingly, the porosity and stiffness of the cell-specific scaffolds were chosen to test the efficacy of a model for the mechanics that views a capillary as a tunnel through a gel^13^,^14^,^15^. By flowing human blood and oxygenated fluid through it, it was established that these constructs support the forces associated with blood flow, and nutrient gradients similar to those measured *in vivo* in a *bona fide* human capillary, validating this model for the mechanics.

This is an important test of the prospects for using LCL as a method for tissue engineering. The MFUs in major organs all incorporate capillaries and so this aspect of the cytoarchitecture must be essential to the physiology and represents a first step in the creation of an MFU. However, the complex architecture makes a capillary difficult to engineer repeatably and precisely. Advanced microscopy has revealed its cytoarchitecture^16^. The lumen of a capillary measures only about 3–30 μm in diameter—so small that erythrocytes (red blood cells) pass through in single file—and it is enclosed by a monolayer of endothelial cells (ECs) that rests on a basement membrane (BM) invested abluminally with pericytes. What is new here is the precision of the cell placement, the control of the cytoarchitecture, and the cell-specific scaffolds, delivered by LCL, which may all be instrumental in the development of MFUs. That is not to say that tubular structures resembling capillaries have not been formed *in vitro*. For example, in tube formation assays, which are widely used as *in vitro* models for angiogenesis, primary or immortalized ECs, mixed with conditioned media and plated on basement membrane matrix, self-assemble into tubes in about 3–12 hr^17^, but there is little prospect for creating an MFU this way. On the other hand, LCL is appealing for engineering MFUs because it offers precise control of the cytoarchitecture. As a final illustration of how this precision could be exercised and harnessed to study biology or pathology, cancer cells were repeatedly trapped in synthetic capillaries. This naive model is used to illustrate the advantages and shortcomings of the LCL-approach to tissue engineering.

## Results and Discussion

### Cell Viability

**Figure 1:**
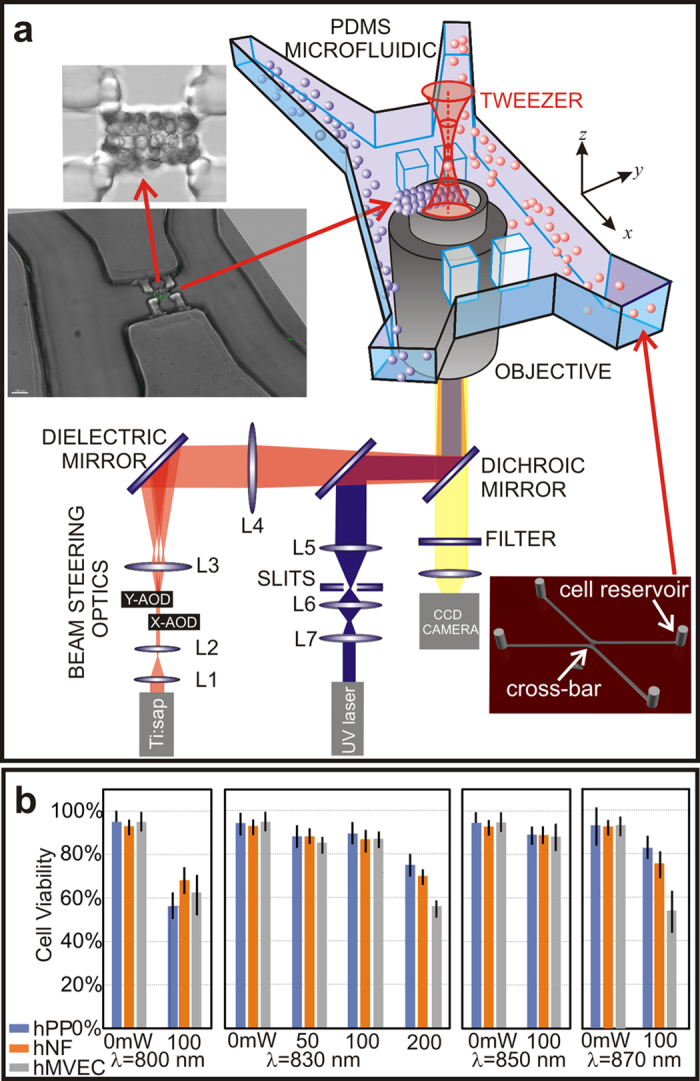
Schematic of a time-shared optical trapping apparatus and microfluidic conveyer used to form a synthetic capillary. (**a**) Cells were manipulated using optical traps formed with infrared light (red path) from a Ti:sapphire laser through a high NA commercial optical microscope. The same optics were used for imaging (yellow path) and near-UV exposure to photopolymerize the hydrogel scaffold (blue path). Two or more cell types were conveyed to the assembly area using the microfluidic network. A hydrogel scaffold was formed in the cross-channel. (**a**, upper insets) micrographs of a 3-layer structure of hMVECs organized using tweezers to resemble a capillary and then encapsulated in a hydrogel scaffold. **(a**, lower inset) perspective rendering of the entire microfluidic device. (**b**) Bar-graphs are shown that relate the viability scores for various cell types 10 h after manipulation with optical tweezers and encapsulation in hydrogel. Three hundred eighteen hMVECs (gray), 246 hNFs(orange), and 438 hPPs (blue) were assayed after exposure. They were exposed to time-shared optical traps at wavelengths of 800, 830, 850 and 870 nm with 0, 50, 100 and 200 mW per cell. The cells were held in the trap for 30 sec prior to gelling in a PEGDA hydrogel and then viability was assessed with LIVE/DEAD stains.

The use of optical tweezers for manipulating living cells offers precise control over the size, shape and composition of the capillary cytoarchitecture (Fig. 1a). However, the concomitant photodamage could compromise cell viability. To avoid absorption, light in the near-infrared (IR) band was employed for tweezing. To assess the effect of the IR radiation wavelength, power and duration of exposure on cell viability, the three cell types used to create a capillary: human microvascular endothelial cells (hMVECs); human pericytes from the placenta (hPPs); and human neonatal fibroblasts (hNFs) were exposed to a time-shared 3×3 array of (nine) optical traps. In pursuit of an optimal wavelength and power, the traps were formed at wavelengths λ = 800, 830, 850, and 870 nm with time-averaged optical power ranging from 0 to 200 mW using a dwell time of 20 μsec over each of nine traps with the duration of exposure to the beam limited to <30 sec. Subsequently, the cells were partially encapsulated in 5.0 kDa PEGDA/PEG-RGDS hydrogel along with dark controls. To promote viability, fresh media flowed through the arrays continuously at a rate of 0.05 μl/min.

The viability scores from this assay remained consistently high (~90%) in the band 830–850 nm for all the cells as long as the time-averaged power ≤ 100 mW; no significant differences were observed between the dark controls (≥ 90% viable) and the number of viable cells after exposure to the trap beam (Fig. 1b). (Likewise MDA-MB-231 breast cancer cells were tested under similar conditions, which has already been reported elsewhere^18^). The viability was especially sensitive to handling, however (see Methods). Thus, with proper handling, cells trapped at λ = 830 nm for <30 sec in a time-shared trap with a total time-averaged power ≤100 mW remained viable.

### Hydrogel Nanomechanics and Composite Scaffolds

Operationally, multiple laminar fluid flows in a four-port microfluidic device were used to convey cell suspensions to a cross-connecting channel where multiple optical tweezers (Fig. 1a, red optical path) were used to pluck them from the flow and assemble them on a hydrogel scaffold. The hydrogel scaffold that partially encapsulated each cell was formed by photopolymerizing a pre-polymer mixture in the cell suspension. Depending on the cell-type, three different hydrogels were formed from either PEG or gelatin, which were modified with either acrylate or methacrylate moieties, and then cross-linked using free radicals generated by exposure of a photoinitiator (PI) to near-UV light (Fig. 1a, blue optical path). These choices for hydrogels were dictated mainly by a model for capillary mechanics in which the interstitium provides significant mechanical support^13^,^14^,^15^.

Due to the high water content, PEGDA and Gel-MA hydrogels possess physical properties that resemble soft tissue. PEGDA scaffolds, in particular, are used prevalently for tissue engineering^19^. They are biocompatible, and the porosity and mechanical properties can be easily tailored. One motivation for choosing Gel-MA was that it naturally incorporates cell-binding motifs like matrix metalloproteinase (MMP), a major factor in angiogenesis, along with RGDs and elastin, but it is soft^20^. On the other hand, PEGDA hydrogels are especially advantageous because they can be made stiffer and hybridized with proteolytic oligopetpides or growth factors to promote specific cell interactions, preservation and degradation^19^,^20^,^21^. In this work, RGDS (arginine–glycine–aspartic acid—serin) and MMP pendant groups were hybridized separately to PEGDA to enhance cell viability, promote adhesion and degradation.

Importantly, the main function of a capillary is not to perfuse blood, but rather to exchange oxygen and other nutrients for waste products between blood and the surrounding tissue. Thus, the mechanical properties—i.e. the volume, porosity and stiffness—have to be commensurate with these functions. Material penetrates through gaps between the interjoined ECs, but is impeded by the BM. The flow of blood and plasma through a capillary is driven by pressure, balanced by viscous forces. Fung *et al.*^13^ have modeled the mechanics of a capillary three ways. According to one model, if a capillary is represented by a tube floating in interstitial liquid, then the high transmural pressure is accommodated by the endothelium and the BM. Since the BM has a modulus ranging from 0.7–10 MPa, it alone could support the tension in the wall^13^,^14^. On the other hand, if the total pressure is accommodated by the whole vessel wall then, assuming it is a gel-like material, its modulus would be only about 100–400 kPa, depending on the thickness. Alternatively, a capillary can be viewed as a tunnel through a gel, and the distensibility related to the ratio of the modulus of the tube wall and its surroundings^13^,^14^. Measurements of the capillary distensibility indicate that the effective modulus of the gel surrounding the tunnel would be 15 kPa^14^. Thus, according to this model, the interstitium could provide significant support for the capillary.

Although the bulk mechanics of hydrogels^22^,^23^ and cells^24^,^25^ have been studied separately, the nanomechanics of hydrogels incorporating cells have not. To gauge the stiffness, the elastic modulus (*E*) was measured by nano-indentation performed on a voxel with an atomic force microscope (AFM). The size of a voxel was determined by the volume proportion between cells and the ECM *in vivo,* which was supposed to be constant for each tissue^26^,^27^. The measured ratio of ECM:cells in each voxel was maintained at 1.1 ± 0.1:1. Infinitesimal strain theory relates the indentation force to the elastic modulus through the relation: 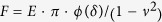, where *E* denotes the elastic modulus, *v* the Poisson ratio, and 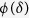 is a function of the indenter geometry (see Methods, supplement and Fig. S1)^28^. Two different probes were used: one was a blunted conical silicon tip of 7–10 nm radius (designated as the “sharp tip”, Fig. S2a left, upper inset); and the other was a tip modified by rigidly attaching a gold microsphere with a 1.5–3 μm diameter (designated as the “gold tip”, Fig. S2a left, lower inset). Regardless of the tip and the voxel size and shape, the data consistently showed a linear relationship between the indentation force and the function *ϕ* (Fig. S2a–c, left) as expected. However, the sharp tip consistently showed a much higher modulus compared to the gold tip (Table 1).

The disparity between the moduli obtained from the same voxel with different tips likely indicates defects in the gel network on a micrometer-scale. Micro-domains of variable stiffness like these have been observed before and attributed to so-called spatial gel inhomogeneities due to the cross-link density distribution^29^. According to elasticity theory, the modulus is supposed to be proportional to the absolute temperature and the cross-linking density, so that 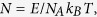 where *N*_*A*_ is Avogadro’s constant, and *k*_*B*_ is the Bolztmann’s constant. Thus, the data implies that the cross-linking density changes about 30-fold in PEGDA when the deformed volume changes from ~1000 nm^3^ to ~1 μm^3^. Likewise, for Gel-MA, there must be a corresponding 5-fold change in cross-linking density. Moreover, the cross-link density was about 10-fold higher in PEGDA compared to Gel-MA.

Interestingly, the mechanical properties of voxels incorporating cells can reflect either the cell or the gel modulus, depending on the scale of the measurement (Table 1). The apparent elastic modulus for a voxel including an hMVEC was determined to be *E*_*PEG*+*cell*_ = 110 ± 20 kPa (Fig. S2c, left) using a sharp tip, which seems to reflect the PEGDA modulus described above, not that of an EC. In contrast, indentation with a gold tip revealed a modulus of *E*_*PEG*+*cell*_ = 4.0 ± 0.9 kPa, similar to other ECs measured alone^24^ or just PEGDA measured with a gold tip. Likewise, the modulus of a Gel-MA voxel incorporating an hNF measured with a sharp tip was *E*_*Gel*+*cell*_ = 35 ± 1 kPa, which was similar to that of a fibroblast^25^, whereas the stiffness measured with a gold tip was only 2.0 ± 0.6 kPa, which was more indicative of Gel-MA. These observations were relevant in the construction and function of a capillary in several ways. When the gel network equilibrates under a mechanical constraint imposed by the surrounding cytoarchitecture, there will be an anisotropic, inhomogeneous distribution of molecules, including water. Calculations show that, near the hNF-Gel-MA interface, the stress may be high and the concentration of water molecules will be greatly reduced within a ~1000 nm^3^ volume^30^. On the other hand, near the hMVEC-PEGDA interface, the concentration of water will not be so affected because the gel swells less when the network is more densely cross-linked.

A lower modulus implies a sparser cross-link density in the gel and higher diffusivity through it. To corroborate this assertion, the mesh size was inferred from the swelling ratio *Q = W*_*eq*_/*W*_*dry*_ obtained by weighing a voxel to determine equilibrium weight (*W*_*eq*_) and dry weights (*W*_*dry*_)^31^. To achieve sub-picogram resolution, the weight was inferred from the shift in the resonant frequency of an AFM cantilever that occurs when the cantilever was loaded with a voxel. When cantilevers were loaded with PEGDA or Gel-MA, a shift in the resonant frequencies of Δ*f*_*dryPEG*_ = −0.29 ± 0.02 kHz and Δ*f*_*wetPEG*_ = −2.60 ± 0.05 kH, and Δ*f*_*dryGEL*_ = −6.2 ± 0.1 Hz and Δ*f*_*wetGEL*_ = −67 ± 9 Hz were observed (Fig. S2a,b, right). The corresponding porosity, 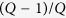, which was estimated to be 90.1% and 90.7% for PEGDA and Gel-MA respectively, factors into the effective diffusion coefficient through the porous medium. To verify this contention, the diffusion of a surrogate protein, fluorescent streptavidin, was also tracked through the hydrogels using confocal microscopy (see supplement and Fig. S3a,b). Thus, with these scaffolds, the modulus of the endothelium could be engineered to be locally much stiffer (>100 kPa) than the interstitium (<40 kPa) as it supposedly is in a *bona fide* capillary^13^,^14^ while still allowing for nutrient and protein diffusion.

### Creating a Capillary with LCL

**Figure 2:**
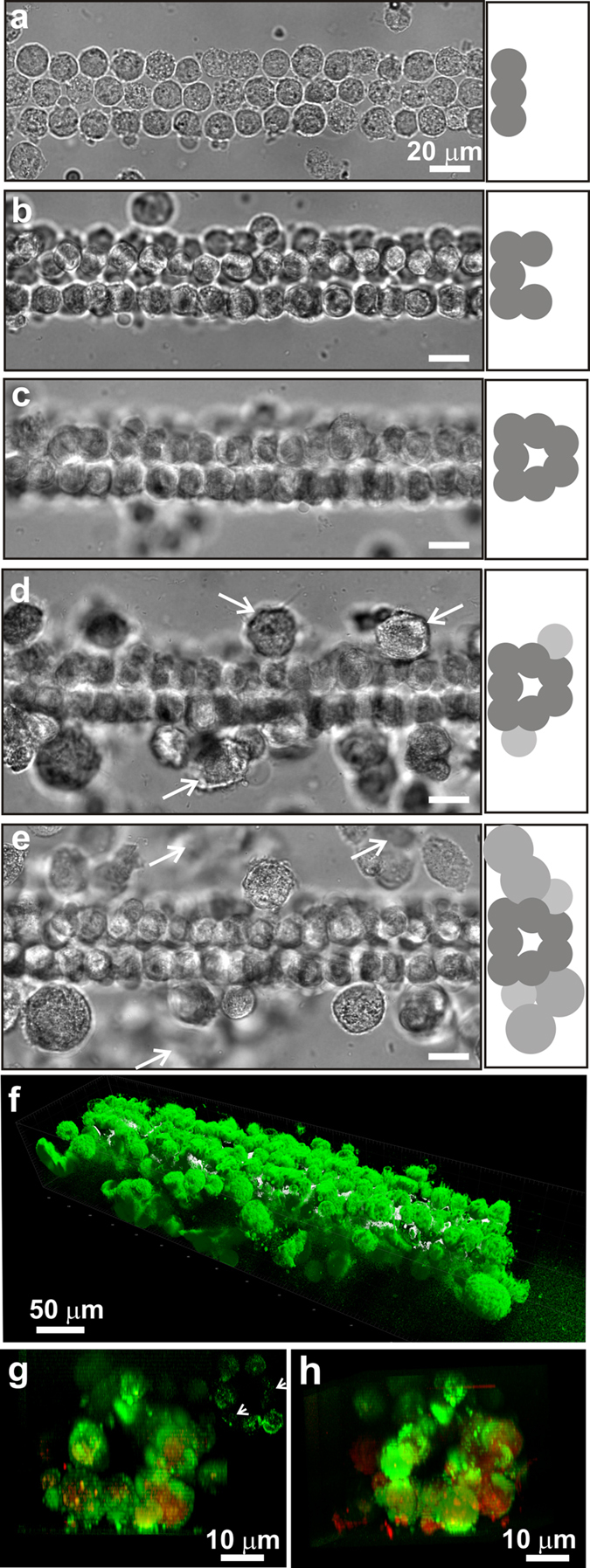
Layer-by-layer assembly of a synthetic capillary. **(a–e**, left) Top-down optical micrographs of a 3-layer structure of hMVECs (endothelials) and hPPs (pericytes) partially encapsulated in PEGDA hydrogel is shown, illustrating the basement layer, the lumen and the attic layer respectively, which was organized using optical tweezers to resemble a capillary about one-quarter millimeter long. The optical micrographs show living voxels each containing a single cell encapsulated in a hydrogel scaffold. Selected hPPs are indicated by the arrows in (**d**), whereas the final capillary encased in voxels constituted from hNFs (fibroblasts) in a Gel-MA scaffold are represented in (**e**) with selected hNFs indicated by the arrows. (**a–e**, right) Schematic representations are shown of the construction with hMVECs denoted by dark gray circles, pericytes by light gray and fibroblasts by medium gray. (**f**) A false-color perspective shadow projection is shown, which was reconstructed from volumetric data acquired from a series of confocal images from IF stained cells to differentiate hMVECs from hPPs and hNFs. As the fluorescent intensity is mapped using shadow projection, the fluorescent surface is visually impenetrable. (**g,h**) Two 20 μm thick slabs are shown, which were reconstructed from volumetric (confocal) data showing cross-sectional (through the lumen) views in the same capillary. To visualize the intraluminal surfaces, the hMVECs were positively stained for CD31 (green), whereas the hPPs and hNFs were positively stained for NG2 (red). (**g**, upper right inset) A thin 0.5 μm cross-section through the same capillary, stained with an FITC-conjugated anti-CD31 antibody, is shown. Cells exhibit localization of CD31 predominantly on the cell surface, whereas localization out of the plane (arrows) was not obvious.

To determine if these mechanics could support blood flow, hMVECs, hPPs and hNFs were assembled layer-by-layer on cell-specific scaffolds into an architecture reminiscent of a human capillary in the cross-channel spanning the gap between the two microfluidic channels (Fig. 1a). Generally, the basement layer was formed from a contiguous 3×30 bed of voxels comprised of hMVECs and PEGDA-RGDS that, in turn, were flanked on either side by voxels comprised of hPPs in PEGDA-RGDS, at a ratio of 10:1 (hMVECs:hPPs) as found *in vivo* (Fig. 2a)^32^. Subsequently, the microscope focus (used for the tweezers and photopolymerization) was shifted along the optic axis 25 μm above the basement layer using a piezo-drive, and then a second (interior) layer, consisting of two 1×30 columns of hMVECs with a gap 5–20 μm wide between them, was formed to create the lumen of the capillary (Fig. 2b). Again, the abluminal volume was flanked by hPPs voxels that reflect the ratio found *in vivo*. Similarly, a third, “attic” layer with the same constituency of cells was assembled from a 2×30 column of hMVECs voxels situated directly above the cells in the second layer (Fig. 2c). Finally, the synthetic capillary was flanked by voxels comprised of hNF cells in Gel-MA, at a ratio of 1:5 to (hNF:hMVECs) to capture the constitution of the interstitium^33^ as they likely play a key role in morphogenesis^33^,^34^. The total time to create a capillary this way, including loading the sample into the microfluidic, stabilizing the flow in the microfluidics, the step-and-repeat lithography, ranged from 30 to 90 min, depending mostly on the capillary length and the cell flux.

In an actual capillary, the endothelium forms the lining of the intraluminal surface and thus, the inter-endothelial contact controls the transport of solutes and circulating cells into the interstitial tissue and vice versa. The hMVECs express at least two transmembrane molecules specific for inter-endothelial contacts: PECAM-1 (platelet endothelial cell adhesion molecule-1 or CD31) and vascular endothelial cadherin (VE-cadherin). To visualize the intraluminal surfaces, the hMVECs were positively stained for CD31 (Fig. 2f–h). Likewise, the hPPs and hNFs were positively stained for NG2 (chondroitin sulfate). Reconstructed volumetric data obtained from immunofluorescence (IF) of the hMVECs, hPPs and hNFs labeled *in situ* showed a continuous capillary (Fig. 2f) and an unobstructed lumen with a 140 ± 43 μm^2^ cross-section about a quarter-millimeter-long (Fig. 2g,h), corresponding to a median diameter of 13.2 ± 2.5 μm^2^ that is comparable to *in vivo* estimates for the diameter of normal human nail-bed capillaries that range from 5 to 12 μm^35^. Capillaries up to a half-millimeter-long (Fig. S5a-e) were produced this way, corresponding to the average length of a human capillary, which is between 0.4 and 0.7 mm^36^.

The endothelial phenotype has been classified as continuous, fenestrated or sinusoidal^37^,^38^,^39^,^40^,^41^,^42^. The ECs comprising continuous capillaries form an uninterrupted lining with tight junctions between them allowing only water and ions to pass through their intercellular clefts, whereas fenestrated capillaries are more porous and allow a limited number of large molecules (proteins) to diffuse through to the interstitium and finally, sinusoidal or discontinuous capillaries exhibit large openings in the endothelium that allow blood cells and plasma to pass through. The cells comprising these synthetic capillaries generally exhibited localization of CD31 on the cell surface predominately (e.g. Fig. 2g, upper right inset). Using specific surface markers (e.g. CD31, ZO-1 and plakoglobin) and cell morphology, it was observed that cell adhesions between adjoining ECs were not tight, however. The gaps observed between adjacent hMVECs likely allow for fluid exchange between the plasma and the surrounding tissue (see the next section). Thus, the cytoarchitecture of these constructs was reminiscent of either a fenestrated or sinusoidal capillaries.

In contrast with LCL, the growth of new blood vessels by angiogenesis can be slow (>7 da)^43^,^44^,^45^,^46^,^47^,^48^,^49^,^50^,^51^,^52^,^53^. To accelerate vessel formation, *in situ* vasculogenesis has been used, which is the spontaneous formation of undifferentiated ECs into blood vessels. Alternatively, pre-vascularized tissue, formed by either angiogenesis or vasculogenesis in 3D gels^49^,^50^,^51^,^52^,^53^ or on patterned surfaces or by 3D printing^7^ can shorten the time to vascular perfusion. Finally, tube assays in which human ECs and pericytes co-assemble form tubular networks in 12 h or less^17^,^50^. However, to our knowledge, there has been no report of mature capillaries with tight junctions forming this way. Vasculogenesis results from a sequence of events mediated by cell–cell and cell–matrix interactions and flow conditions, but the mechanisms of maturation and specialization of microvasculatures have yet to be elucidated^49^,^50^. In all of these approaches, the cells, cues and micro-scale flow play key roles in morphogenesis^50^. It should be possible to discover the mechanisms underpinning maturation of a microvasculature by tracking remodeling in degradable hydrogels under suitable culturing conditions.

It has been hypothesized that exposure of ECs to mechanical forces associated with blood flow and growth factors will affect integrin-, Rho GTPase- and SRF-dependent signaling that control vessel remodeling and produce a mature endothelium, but it remains untested^50^. To test this hypothesis, systematic strategies will have to be employed involving the utilization of different cell combinations with cell-specific ECMs that promote remodeling and morphogenesis, such as PEGDA hybridized with recognition motifs such as MMP, along with different GF-supplemented media and mechanical forces to produce a mature endothelium. This can be accomplished in a synthetic capillary.

**Figure 3:**
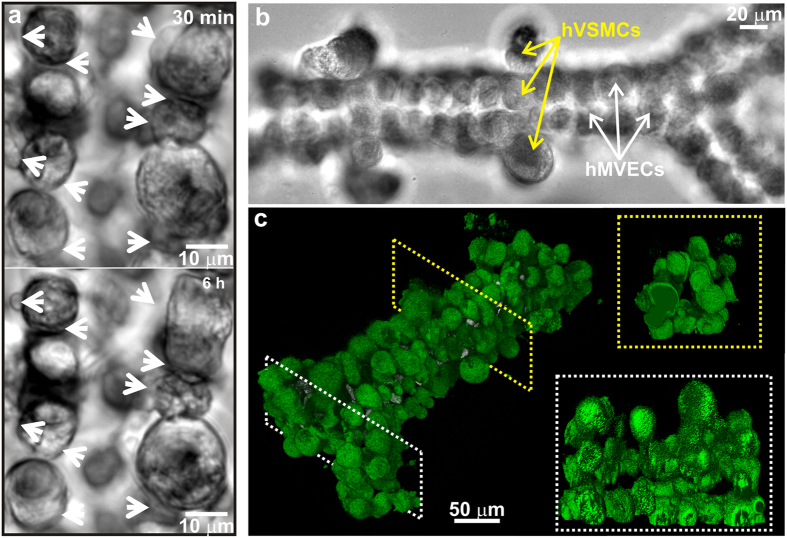
Remodeling in a capillary and a branched arteriole created with LCL. (**a**) Time-lapse optical micrographs are shown, which were taken every 30 min show remodeling in a synthetic capillary. In particular, the images of the lumen acquired at 30 min and 6 h are shown. The white arrows, which are in fixed positions in the two images, highlight areas where cells are remodeling. (**b**) A top-down optical micrograph of a multi-layer structure of hMVECs and hVSMCs, partially encapsulated in PEGDA/PEG-RGDS hydrogel, is shown, which was organized using optical tweezers to resemble a branched arteriole about 0.33 mm long. Selected hSMCs and hMVECs are indicated by the arrows. (**c**) A false-color perspective shadow projection is shown, which was reconstructed from volumetric data acquired from a series of confocal images from immunofluorescently stained CD31-postive cells or the arteriole in (**b**). (Top and bottom insets) Cross-sectional views (through the lumen) of the arteriole in (**b**) before (top) and after (bottom) the bifurcation are shown, which are positioned as indicated by the yellow and white dotted rectangles on the left, respectively.

To illuminate the prospects, synthetic capillaries were created with the same methods illustrated in Fig. 2, but using a degradable PEGDA-MMP scaffold for the hMVECs instead of PEGDA-RGDS, and then the time progression of the cell morphology was tracked with confocal microscopy for up to 12 h with images acquired at regular 30 min intervals with and without whole human blood flowing through them (Fig. 3a). Remodeling of the endothelium was evident within 6 h (white arrows)—in some cases, cells pressed tight against each other to form flat broader junctions while in other cases protrusions sprouted from the cells (Fig. 3a). It was also observed that the hMVECs forming the lining become polarized when subjected to shear force associated with human blood flow under differential pressures up to 1 kPa for up to 24 h, (Fig. S4). Using established protocols^54^, the polarization of the cells was tracked by following the position of the Golgi apparatus stained with BODIPY-FL-C5-ceramide. Confocal microscopy revealed the localization of the Golgi apparatus in hMVECs (white arrows, Fig. S4) near the lumen that constituted the 2^nd^ layer of a synthetic capillary, indicating the onset of polarization of hMVECs. Although force is only one element driving maturity, this preliminary evidence indicates the prospects for testing hypotheses regarding remodeling and maturation of the endothelium.

As another illustration of the versatility and precision of the tool, constructs that resembled *branched* arterioles (in size) consisting of hMVECs and human vascular smooth muscle cells (hVSMCs) were also produced using LCL (Fig. 3b,c and S5 f-h). To visualize the intraluminal surfaces, the hMVECs were positively stained for CD31 and likewise, the hVSMCs were positively stained for CD130a (PDGFRα). Reconstructed volumetric data obtained from CD31-stained hMVECs showed a continuous bifurcated capillary (Fig. 3c) and an unobstructed lumen with a 616 ± 39 μm^2^ cross-section, branching into two vessels; one with a 232 ± 40 μm^2^ cross-section and another with a 365 ± 49 μm^2^ cross-section. Creating these types of structure is especially important because *in vivo* adhesion depends on the anatomical, geometrical and physiological fluid flow conditions as well as the interactions with the endothelium. In particular, bifurcations disturb blood flow, which predisposes them to higher adhesion relative to a straight channel^55^.

### The flow of blood and nutrients (oxygen)

**Figure 4:**
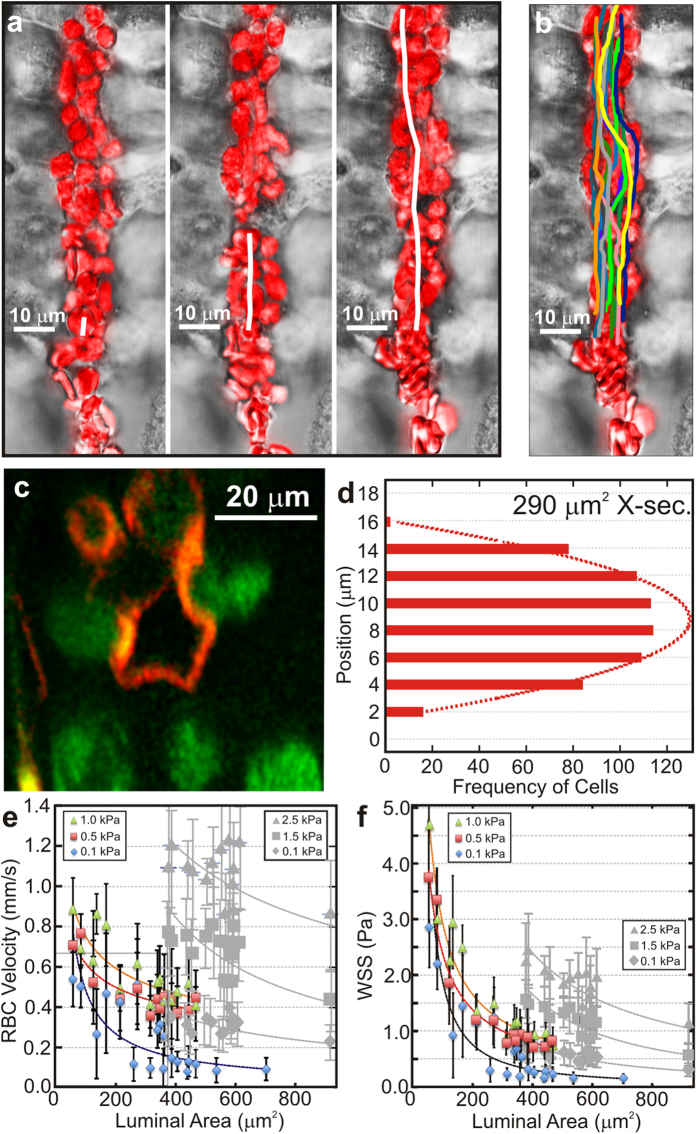
Erythrocytes (red blood cells) perfusing through a synthetic capillary. (**a**) False color representation of erythrocytes flowing through a capillary are shown, which were obtained from a series of confocal slices through the lumen of the vasculature acquired every 16 ms. These snapshots shown here were taken at 48 ms intervals. The erythrocyte trajectory is indicated by the white solid line. In this case, the velocity of the cell, estimated from the distance an erythrocyte traveled between frames, was 586 ± 6 μm/s. (**b**) Like (a), but a superposition of the positions of ten erythrocytes as they flow through the capillary. (**c**) A confocal slice through the vasculature like that shown was used to estimate the cross-sectional area of the lumen. Fluorescent microspheres 40 nm in diameter were used to define the lumen as a function of position along the axis of the capillary. (**d**) Histogram of the axial velocity versus the position in the lumen is shown, which was obtained from measurements of >1000 erythrocytes. The dotted red line is the best-fit parabola; the deviation is an indication of a non-Newtonian fluid. (**e**) A summary of the measurements of the axial velocity as a function of the cross-sectional area of the lumen is shown, which was obtained for two capillaries: one with a diameter <10 μm at differential pressures of 0.1, 0.5 and 1.0 kPa (color); and another with a diameter <20 μm at differential pressures of 0.1, 1.5 and 2.5 kPa (grey). The lines, which represent power-law fits to the data, act as guides to the eye. (**f**) Like (**e**), but a corresponding summary of the wall shear stress (*WSS*) on the same capillaries as a function of the cross-sectional area inferred from the axial velocity with pressure as a parameter.

The role blood flow, perfusion and local oxygen consumption play in this construct had to be tested to prove out the model. Blood is a non-Newtonian fluid with a viscosity that depends on the volume occupied by the erythrocytes (hematocrit) and decreases with shear rate. To assess how the cytoarchitecture and mechanical properties affect the hemodynamical flow, a pressure gradient, Δ*P*, ranging up to 2.5 kPa was imposed across the microfluidic channel connecting to the capillary lumens with cross-sections of 56 ± 7 μm^2^ and 457 ± 72 μm^2^ and then 10% washed human erythrocytes in PBS was perfused through them (Fig. 4 and videos V1,V2,V4-V4).

A comparison of a sequence of high-resolution confocal transmission images revealed the axial erythrocyte velocity, *V*_*ax*_, (Fig. 4a,b) corresponding to the local cross-sectional area of the lumen (defined by fluorescent nanospheres that adhered to the adluminal surface after the erythrocytes were forced through it—Fig. 4c). Due to their volume, the erythrocytes avoided the capillary wall and so a histogram of the velocity profile measured with 2 μm resolution transverse to the capillary axis (Fig. 4d) was found to be blunter than a parabola (dotted line) indicative of a Newtonian fluid. In addition, as expected, the velocity gradient or the shear rate near the wall was steeper than that derived from a quadratic velocity profile. The peak axial velocity, which depended on the luminal area and the pressure (Fig. 4e), was comparable to that measured *in vivo* in capillaries and post-capillary venules of the bulbar conjunctiva in the human eye^56^. The wall shear stress (*WSS*), estimated from Newton’s law (Methods), ranged from 0.9 to about 4.5 Pa. A steep, six-fold decline (from the best fit) of *WSS* values as a function of the cross-sectional area was observed, which was also consistent with that found in human capillaries^56^,^57^.

Blood flow through the capillary under these conditions and the concomitant interstitial flow across it exposes the hMVECs to mechanical forces. In particular, the mechanical force exerted by blood and plasma are likely to co-direct vascular morphogenesis, which can cause vessel segments to sprout, dilate, or remain quiescent. However, the minimum shear stress required to attenuate morphogenesis in the presence of VEGF is supposed to be 0.01–0.3 Pa^58^. Therefore, under these conditions, endothelial sprouting was expected to be suppressed.

**Figure 5:**
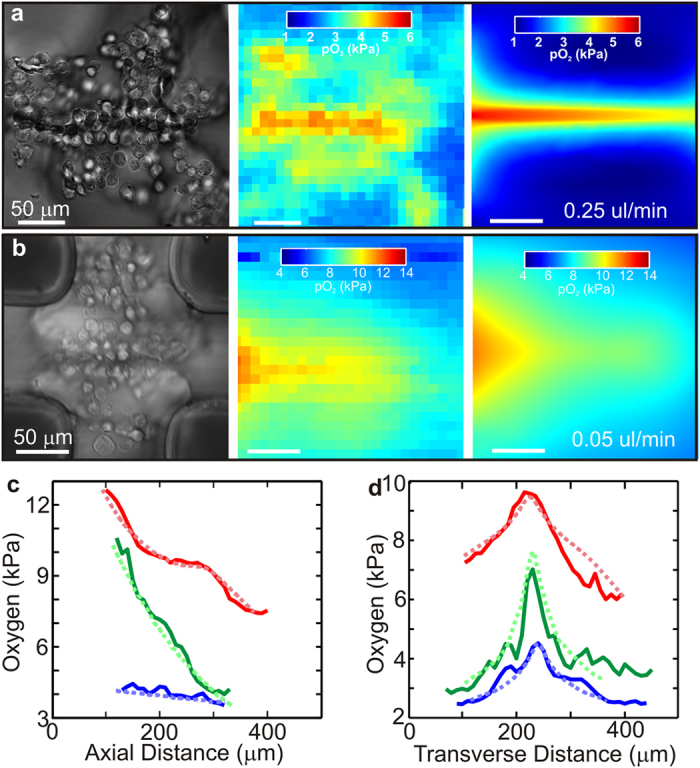
Oxygen flow through and gradients across a synthetic capillary. (**a,b**, left) Tiled transmission images of two synthetic capillaries embedded in a microfluidic device are shown. The lumen was assembled from HMVECs and hPPs, whereas the abluminal cells were hNFs. Rectangular PDMS supports (corners) were used for mechanical support. The capillary in (**a**) has a lumen with a 100 μm^2^ cross-sectional area and experienced a flow of 0.25 μl/min, whereas the capillary in (**b**) had a 400 μm^2^ lumen and was subject to a 0.05 μl/min flow. (**a,b**, center) Reconstructed 2D heat map of the oxygen profile is shown, which was acquired from the structures on the left. The measurements of the oxygen concentration were accomplished in 10 μm steps. (**a,b**,**right**) FESs of the oxygen flow through an idealized capillary under flow conditions similar to that used for the structures on the left is shown, which takes into account, diffusion, migration, and consumption of oxygen molecules by the cells. The partial pressure of dissolved O_2_ across the endothelial cell wall was about 15 kPa regardless of the differential pressure across the capillary. (**c,d**) The oxygen gradients measured along the capillary axes and in the center transverse to them are shown. The (top,bottom) solid lines correspond to measurements extracted from the capillaries in (**a,b**) respectively, the center solid line was associated with a capillary shown in the supplement (Fig. S6). The dotted lines represent the results of FES.

Oxygen is a key nutrient;^59^,^60^ its uptake ranges from 0.3–40 mol·m^−3^·s^−1^ in human tissue. Thus, a viable model also has to accommodate the transport and consumption of nutrients like oxygen. The oxygen profiles in synthetic capillaries were measured directly using the fluorescence from a medium containing a ruthenium compound (ruthenium tris-dipyridyl dichloride hexahydrate—RTDP) and a multi-core optical fiber probe^61^. That gradients in effectors such as oxygen develop along and transverse to the capillary axis can be appreciated from the fluorescent contours and the corresponding optical micrographs of capillaries (Fig. 5a,b, left, center). Clearly, the oxygen concentration diminished with distance along the axis of a >200 μm long capillary in Fig. 5a from the value at the inlet pO_2_ = 4 kPa to pO_2_ = 3.4 kPa at the outlet, which is consistent with uptake from viable cells in the tissue^62^,^63^. On the other hand, with a five-fold reduction in the flow, the oxygen drops from 14.0 to 7.5 kPa (Fig. 5b).

The oxygen tension along the capillary axis (Fig. 5c) can be represented by an exponential decay at a rate of Λ_||_ = 357 (red), 193 (green) and 393 (blue) μm depending on the inlet pressure, the differential and the corresponding flow rate. Independent of the flow rate, a gradient also develops in the interstitial pO_2_ as well, as the transverse distance from the capillary axis increases with an exponential decay rate of Λ_⊥_ = 61 ± 10 μm although it depends critically on the cell density and constituency in the interstices. Nearly anoxic conditions were observed in this tissue construct after 1 hr without flow (Figs. S6,S7), which is nevertheless consistent with the oxygen profile measured in explants^62^.

These data, especially the exponential decay, indicate that the oxygen supply within this engineered tissue was determined by diffusion, advection and consumption. The diffusion coefficient of oxygen in water is *D*_*water*_ = 2,100 μm^2^·s^−1^, whereas the diffusivity in tissue is typically less, ranging about *D*_*tissue*_ = 1,750 μm^2^·s^−1^ 63. Thus, the luminal wall likely reduces the diffusion of oxygen so that the measured transverse gradient reflects, not just consumption, but the cell density as well. Finite element simulations (FES) that account for the diffusivity and consumption of oxygen show that the mass transport of oxygen-rich medium through and around a synthetic capillary was sufficient to maintain physiology (Fig. 5a–d), whereas the absence of flow reduced dramatically the abluminal oxygen supply (Figs S6 and S7). The oxygen gradient was evident from the low oxygen partial pressure (blue) outside relative to the lumen of the capillary and corresponds closely with the measurements (Fig. 5a–d). The diffusivity of nutrients such as oxygen in these constructs seems to be limited by the scaffold—likely the clefts between the hMVECs—not the endothelium. Thus, these tubular micro-channels, constructed from human cells function, much like a fenestrated capillary; they successfully flow human blood (and plasma) and nutrients such as oxygen, but the oxygen diffuses through the endothelial lining.

### Trapping a cancer cell in a synthetic capillary

Taken altogether, these observations support the notion that LCL could be used to engineer tissue with a microvasculature. But how would the tissue be used? To concretely illustrate the advantages and expose some of the shortcomings, synthetic capillaries were created as a naive model to explore the interactions between a breast cancer cell and the endothelium. When it is finally developed, a model like that might be used to study the first steps in extravasation that occur during metastasis. Metastases are formed by cancer cells that break away from the primary tumor, travel via the circulatory system, and become lodged in capillaries at a secondary site^64^,^65^,^66^,^67^,^68^,^69^. Once lodged in a capillary, cancer cells escape or extravasate to the surrounding tissue. The first two steps in the extravasation of cancer cells through a capillary endothelium into the organ are rolling and adhesion of the cancer cell in the capillary^64^.

**Figure 6:**
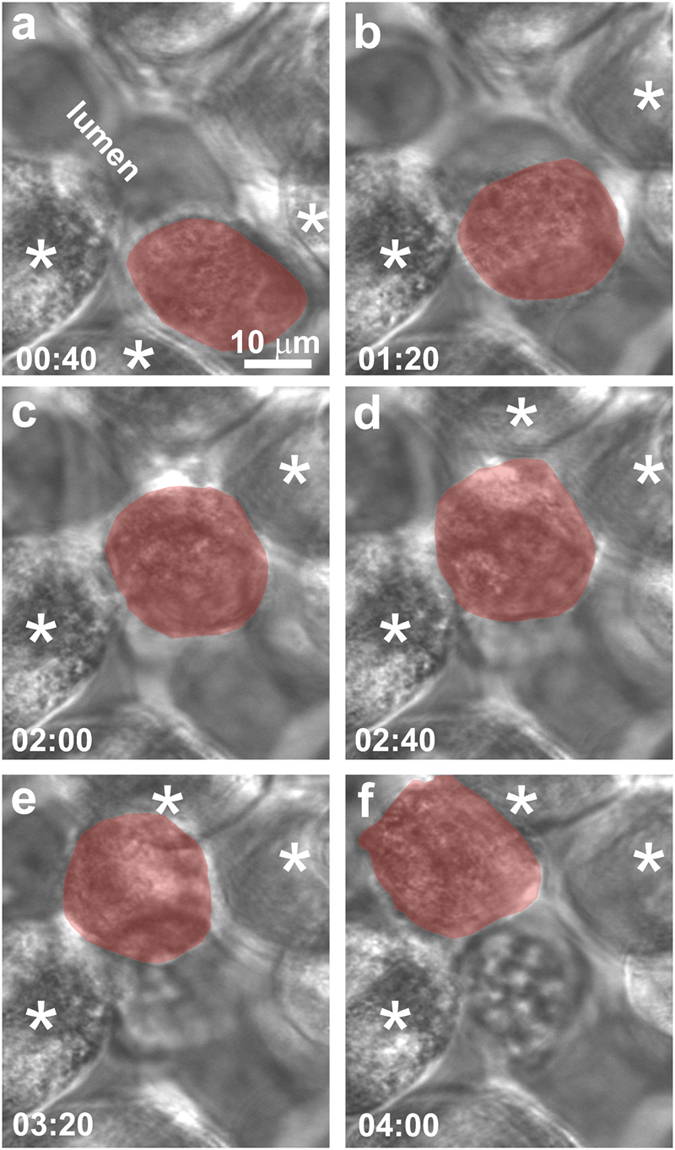
A metastatic human breast cancer cell trapped in a synthetic capillary. Time-lapse optical micrographs taken every 10 min are shown, which illustrate the progress of a single MDA-MB-231 cancer cell (highlighted in red) over 4 h. The hMVECs that contact the cancer cells are indicated with asterisks.

LCL was used to create capillaries with lumens graded in cross-section to trap metastatic MDA-MB-231 human breast cancer cells (Fig. 6). Subsequently, optical tweezers were used to introduce a single cancer cell into them, and then EC and cancer cell growth media (1:1) were flowed through the microfluidic inlet (0.5 μL/min) to trap the cancer cell in the capillary without forcing it through. The time progression of the cancer cell in the capillary was followed with confocal microscopy for up to 48 h with images acquired at regular 10 min intervals. The progression revealed several interesting features. In eight separate observations, the cancer cell stayed dormant (data not shown). However, a few of the cancer cells were motile against the medium flow, adhering and changing morphology when making contact with neighboring hMVECs (Fig. 6 and the corresponding video V3 in the supplement) without compromising viability.

These observations of rolling and adhesion of a cancer cell in a synthetic capillary are too limited to be conclusive, but the exercise revealed aspects of the model that need development. On the one hand, real-time *in vivo* imaging of extravasation has been accomplished in small animals^65^, which can capture some facets of the complexity of human responses, but fails to capture others that are species-specific^1^. Likewise, it is challenging to develop high-throughput systems to evaluate hundreds of animals. In particular, an accurate model for extravasation should include whole human blood, leukocyte and platelets, since they have been implicated in adhesion^64^.

Among the advantages LCL offered was precise control of the cytoarchitecture and cell placement, and cell-specific ECMs. The constructs used for these tests each consisted of heterogeneous, hierarchically organized populations of <100 human cells that were assembled layer-by-layer in <30 min and remained viable for >48 h. In particular, the cross-sectional area of the lumen, which ranged from 352 ± 102 μm^2^ to 669 ± 142 μm^2^ (Fig. 6) was designed and implemented so that part of the capillary would be just smaller than a typical MDA-MB-231 cancer cell, which was gauged to have an area of 460 ± 190 μm^2^ (*n* = 20 cells). Moreover, the same cytoarchitecture was reproduced repeatedly—eight other capillaries were created with practically identical cross-sections. Finally, whole human blood can be perfused through synthetic capillaries constructed from human tissue, but clotting becomes problematic without an anticoagulant (Video V4).

Even though the number of cells in these constructs was comparable to that comprising an MFU, the superstructure of an organ consists of 10^6^-10^7^ MFUs. Therefore, the throughput associated with LCL implemented this way was inadequate to the task of creating whole organs. However, the technology required to improve on the time-to-product from 30 min has already been established. Improvements can be made in the microfluidic conveyance to the assembly area to increase: 1. the flux, while avoiding cell aggregation; and 2. the number of optical tweezers used to manipulate cells. Mainly, it requires multiple optical tweezers controlled independently. This requirement has already been satisfied. The number of tweezers can be increased 100-fold—more than 400 cells have been trapped simultaneously^70^. The number is limited by the irradiance and damage threshold of the optics and AODs. Thus, hundreds of cells could be manipulated into position concomitantly under computer-control using image recognition software to acquire cells conveyed to an assembly area using laminar flow in a microfluidic channel. It is only necessary to avoid excessive shear on the cells.

In contrast with a synthetic capillary, other *in vitro* methods that test the ability of cells to migrate on or through a solid substratum such as scratch assays, trans-well migration assays, and invasion assays, do not recapitulate the anchorage independence required for metastatic dissemination through the circulation^68^. Moreover, invasion assays use only a single type of ECM, which fails to account for the differences in the ECM composition of the primary and secondary sites^68^,^69^. Therefore, an *in vitro* model consisting of human cells in the proper social context with cell-specific scaffold is needed to properly analyze the steps in extravasation and early metastasis. Synthetic capillaries could play this role, but the constructs produced so far have been immature without tight junctions.

## Conclusion

In summary, it has been established that LCL can be used to precisely control the type and position of cells on a composite, cell-specific hydrogel scaffold to create microstructures that functionally resemble a fenestrated or sinusoidal blood capillary with a nanostructure consistent with the elastic modulus and porosity of human connective tissue. In particular, with these scaffold, the modulus of the endothelium was engineered to be locally much stiffer (>100 kPa) than the interstitium (<40 kPa) as it supposedly is in a *bona fide* capillary^13^,^14^, while still allowing for nutrient and protein diffusion. These constructs supported the forces associated with human blood flow, and nutrient gradients similar to those measured in a *bona fide* human capillary.

The application of LCL to tissue engineering is compelling for several reasons. First, it has a facility for precisely and repeatedly placing human cells in a specific cytoarchitecture. A capillary was chosen for illustration because it forms the basis for many MFUs, but in principle any tissue architecture consisting of a heterogeneous population of 100–1000 cells could be organized hierarchically this way to express a predictable function. With model microvasculatures such as a bifurcated arteriole (Fig. 3b,c) or an under-sized capillaries (Fig. 6), cell adhesion, which is supposed to be highly dependent on the anatomical, geometrical and physiological fluid flow conditions, can be tested. Models like these, which can be repeatedly produced, could be important for drug discovery. Second, the precision of the cell placement is submicron. This is especially important in the construction of a capillary because the lumen is so small (3–30 μm in diameter). So far, the literature has mainly focused on larger diameter (arteriole-sized) vessels that were self-assembled without the prospect for larger scale hierarchical organization. Third, not only can cell placement be controlled with precision, but so can the ECM. With LCL it is possible to tailor the microenvironment of each cell-type by photo-polymerizing a specific pre-polymer in each voxel to form cell-specific scaffolds (Fig. 2). Thus, the volume, stiffness, porosity, adhesion, and degradation in the ECM can be controlled with single cell precision. Finally, a complex hierarchically-organized cytoarchitecture can be assembled and perfused in as little as 30 min, which contrasts with angiogenesis that can take a week before structures can be perfused. No other method for tissue engineering offers these features.

## Materials and Methods

### Cell Culture

All the cells were cultured and harvested similarly.

#### Human microvascular endothelial cells (hMVECs)

The hMVECs (CC-2543, Lonza) were cultured in endothelial growth medium (EGM, Lonza), according to the manufacturer’s (Lonza) specifications. Briefly, the hMVECs were maintained at 37 °C, in a humidified incubator at 5% CO_2_/ 95% air until they reached 70–80% confluency (6–8 da after seeding). Only cells at passage numbers 4–7 were used in this work. These confluent hMVECs reproduced the growth-arrested, quiescent state of normal blood vessels *in vivo*, expressing vascular endothelial (VE)-cadherin at cell–cell junctions. Subsequently, the medium was aspirated, the adherent hMVECs were then washed with 30 mM HEPES-BSS buffer (CC-5024, Lonza) and trypsinized by adding a volume of trypsin-EDTA solution (CC-5012, Lonza) sufficient (2.0 mL) to cover the cell surface in the T25 flask and then incubated at room temperature for 2–4 (5) min. Once they appeared rounded and began to detach, the cells were harvested by sharply rapping the culture flask. The reaction was stopped by adding (4 mL) of trypsin-neutralizing agent (CC-5002, Lonza). The detached cells were transferred to a sterile 15 mL tube and centrifuged at an RCF of 200×g for 5 min to pellet the cells. The supernatant was aspirated and the cell pellet was re-suspended in PBS by pipetting gently no more than 2-3 times.

#### Human neonatal fibroblasts (hNF)

Likewise, the hNFs (CC-2509, Lonza) were cultured in fibroblast basal medium (CC-3131, Lonza), according to the manufacturer’s specifications until they reached 90% confluence (5-7 da after seeding). After two passages, the cells were harvested essentially as described above.

#### Human pericytes from placenta (hPP)

Finally, hPPs (C-12980, Promocell) were cultured in growth medium (C-28040, Promocell), according to the manufacturer’s specifications until they reached 70–90% confluence (6–8 da after seeding) and then harvested as described above.

#### Human vascular smooth muscle cells (hVSMC)

The hVSMCs (CC-2581, Lonza) were cultured in smooth muscle growth medium, according to the manufacturer’s (Lonza) specifications. Briefly, the cells were maintained at 37 °C, in a humidified incubator at 5% CO_2_/ 95% air until they reached 70–80% confluency (6–8 da after seeding) and then harvested as described above.

### Microfluidic Device

A multi-channel microfluidic device was used to convey cells to an assembly area. The microfluidic was a four-port, two-channel device with a cross-bar connection between the channels. (Fig. 1(a), lower right inset). The two entry-channels, which were 200 μm wide and 150 μm deep, merged into a single 1.2 mm-long and 600 μm-wide cross-bar laced with four-125×125 μm^2^ pedestals at regular intervals, which were used for mechanical support during assembly. The microfluidic device was formed from poly (dimethylsiloxane) (PDMS) using a mold-casting technique. A master mold of the design was generated by stereo-lithography (FineLine Prototyping, Raleigh, NC) and made of DSM Somos ProtoTherm 12120, a strong, high temperature tolerant plastic. The PDMS silicone polymer used to create the chips was commercially available as Sylgard 184 (Dow Corning), a two-part polymer mix. The two parts were mixed thoroughly at a 1:10 ratio of curing agent to epoxy. The mixture was degassed in house vacuum for 30 min and then poured into the master mold, where it was cured at 75 °C for at least 15 h. After cooling to room temperature, the plastic, which hardened to a rubber-like consistency, was peeled away from the mold yielding the inverse pattern of the master. The microfluidic channels were connected to external pressure and fluid reservoirs through a 2 mm diameter hole at the inlet and outlet ports incorporated in the form.

Though PDMS is transparent, our microfluidic chip was thick (>4.0 mm) and light scattering through it prevented optical access through the top. Accordingly, for optical access, the bottom of the PDMS was sealed with #0, 24×60 mm (Gold Seal) cover glass. To tightly bind the PDMS microfluidic to the cover glass, a covalent bond between the PDMS and glass was formed using an oxygen plasma in a Harrick plasma cleaner (PDS-32G). The oxygen plasma generates silanol (Si-OH) groups on the surface of PDMS, which reacted with silanol groups on the glass surface to form an Si-O-Si bond. The plasma was monitored to ensure a bright, purplish color, and left on for 180 sec. The PDMS chip was gripped by the sides, and placed in contact with the coverslip, and then a uniform pressure was applied (magnetically) at 75 °C overnight to form the bond.

To enhance hydrogel adhesion the internal surfaces of the microfluidic device were treated with a methacrylate silane treatment that crosslinked with the hydrogel. A 2% (v/v) solution of 3-(trimethoxysilyl) propyl methacrylate was made in 10 mL of 95% ethanol. The solution was adjusted to a pH of 5 using 50 μL of glacial acetic acid. 500 μL of this solution was pushed through the microfluidic chip using a 1 mL syringe, and incubated for 5 min at room temperature. The chip was then flushed with ethanol (5 ml) and deionized water (5 ml) and baked in the vacuum oven at 75 °C for 30 min.

### Hydrogel and Photopolymerization

The cells were partially encapsulated in hydrogels formed from either PEG or gelatin precursors that were modified with either acrylate or methacrylate moieties, and crosslinked using free radicals generated by exposure of a photoinitiator (PI), 2-hydroxy-[4-(hydroxyethoxy)]-2methyl-1propanone (Irgacure 2959, Sigma) at 0.3% (w/v) to near-UV light. The hydrogel used to form the endothelium (PEGDA-RGDS) was comprised of PEG diacrylate (PEGDA), and acryl-PEG-RGDS with adhesion ligand RGDS (arginine–glycine–aspartic acid—serin, N-terminus to C-terminus, American Peptide Company) to improve the cell attachment. The peptide sequence (1 mg/ml) was covalently coupled to a mono-acrylated polyethylene glycol of molecular weight 3.4 kDa containing an N-hydroxysuccinimidyl group on one end (Acryl–PEG 3400–NHS, Laysan Bio) by reacting equimolar amounts of Acryl–PEG3400–NHS and RGDS in 50 mM sodium carbonate buffer at pH 8.2 for 2 h at room temperature. Once the reaction was complete, the product was dialyzed against water overnight using dialysis tubing of molecular weight cutoff 1.0 kDa. The dialyzed product was then lyophilized to obtain the monoacrylated PEG with a RGDS sequence (Acryl–PEG 3400–RGDS). Just prior to injection into the microfluidic, cell suspensions were combined 1:1 with a pre-polymer mixture made of 5.0 kDa polyethylene glycol diacrylate (PEGDA, Laysan Bio) at 10% (w/v) in the final solution, 3.4 kDa PEG-RGDS at 0.2 mM; cell media; and PI.

Following other work^21^, to promote remodeling of the endothelium, a degradable PEGDA-MMP was also synthesized using MMP-sensitive peptides by reacting the peptide chain GCRDVPMSMRGGDRCG (GenScript, Piscataway Township, NJ) with the succinimidyl valerate ester (SVA) on an acryl-PEG_2000_-SVA (Laysan Bio Inc., Arab, AL) in 50 mM sodium bicarbonate (pH 8) at a 1:2 mole ratio for 4 h. The reaction mixture was then dialyzed for 24 h to remove unreacted reagents, lyophilized and stored at −80 °C until use.

Finally, methacrylated gelatin (Gel-MA) was used to encapsulate fibroblast on the abluminal surface of the capillary. The Gel-MA was synthesized from porcine skin. Type A porcine gelatin was mixed at 10% (w/v) into Dulbecco’s phosphate buffered saline (DPBS) at 60 °C and stirred until fully dissolved. Methacrylate anhydride (Sigma) was added to the methacrylate 50% of the lysine group at a rate of 0.5 mL/min to the gelatin solution under stirred conditions at 50 °C. This mixture was reacted for 1 h. The fraction of the lysine groups reacted was modified by varying the amount of methacrylate anhydride present in the initial reaction mixture. Following a five-fold dilution with additional warm (40 °C) DPBS to stop the reaction, the mixture was dialyzed against distilled water using 12–14 kDa cutoff dialysis tubing for 1 week at 40 °C to remove salts and methacrylic acid^71^. The solution was then lyophilized for 1 week to generate white porous foam and stored at −80 °C until it was used. Just prior to injection into the microfluidic, cell suspensions were combined 1:1 with the Gel-MA precursor 5–20% (w/v); in PBS; and a photoinitiator, 2-hydroxy-[4-(hydroxyethoxy)]-2methyl-1propanone at 0.5% (w/v). (Irgacure 2959, Sigma).

All the cell-precursor suspensions were mixed well to achieve a homogeneous mixture by slowly pipetting 3–4× and then injected into the microfluidic using a syringe pump through a 23G-gauge needle. The hydrogel was photo-polymerized in the microfluidic using near-UV light from a metal halide light source (X-CITE 120Q, Lumen Dynamics) that was bandpass filtered (λ = 360 ± 12 nm) UV filter (Semrock) at an irradiance of 5.0 ± 0.8 W/cm^2^. The pre-polymer-cell suspension was exposed to the near-UV light for 400 ms. A rectangular mask was placed in the UV path to control the size and shape (15×15 μm^2^) of the hydrogel at the objective focus. Immediately after each microarray was formed, the cell array was flushed with fresh pre-polymer mixture. At the end of the lithography, the microfludic was flushed with fresh culture medium.

### Live Cell Lithography

Cells in the assembly area in the cross-bar of the microfluidic device were organized into 3D, heterogenous arrays using multiple optical tweezers. The tweezers were formed using a Zeiss Fluar 40× oil immersion objective (1.3 NA) in a Zeiss Axio Observer inverted microscope with light from a tunable, CW Ti:sapphire laser (3900S, Spectra Physics) operating at λ = 830 nm, pumped at 532 nm by a 15 W Nd:YVO4 diode-pumped solid state laser (Millenia Pro15J, Spectra Physics) as described elsewhere^11^,^18^,^70^. To create multiple, time-multiplexed tweezers, a single laser beam was deflected transverse to the direction of propagation using two orthogonally mounted acousto-optic deflectors (AOD, AA-Optoelectronic), optimized for maximum diffraction efficiency at the wavelength of interest (860 nm), to give independent control of the *x-* and *y-*positions of a beam, allowing for the creation of a two-dimensional (2D) array of traps. The AODs were used to create 2D, 3×3 arrays of time-multiplexed optical tweezers that were used to pluck cells from a laminar flow and steer them into the array. The time-averaged power in each trap within the array was typically <8 mW. Laser power was recorded as the time-averaged power based on the duty cycle (i.e. the number of traps) in the time-shared array.

The laser beam was scanned rapidly from one trap to the next in the 2D array, dwelling at the desired trap position just long enough to illuminate it and fix the location of a cell there. When the AOD deflected the beam to the next trap location in the array, cells that were not illuminated diffused from the target locations and were dispersed. To prevent dispersal, the rate of deflection between traps was carefully chosen relative to the time the cell spent in the dark. Typically, the light/dark time for each cell was 20 μs/ 100 μs. The cellular arrays were not limited to planar configurations; the optical tweezers could be offset along the optic axis using a computer-controlled piezo-stage upon which the microfluidic device rested as an elevator. Once it was in position, a cell was partially encapsulated in hydrogel. Handling one cell every 5–20 sec was typical. Following this approach, capillaries were assembled voxel-by-voxel, layer-by-layer. When a capillary was completed, the cell suspension was flushed and a fresh volume was introduced into the microfluidic to avoid prolonged exposure to free radicals.

The microfluidic device, along with the entire Zeiss Axio Observer inverted microscope, was encased in an environmental chamber (XL S1, Pecon/Zeiss, Switzerland). After assembly, to maintain the tissue, the chamber was used to automatically regulate using feedback: the temperature (37 ± 0.5 °C), the partial pressure of CO_2_ (pCO_2_ 5% atm). To establish the optimal wavelength and power for tweezing cells, viability was tested by partially encapsulating them in hydrogel (along with dark controls) and assaying them 10 h later with green-fluorescent calcein-AM, which indicated intracellular esterase activity, and red-fluorescent ethidium homodimer-1 that assessed the plasma membrane integrity (Molecular Probes, C3100MP).

### Testing the Mechanical Properties of the Hydrogel with AFM

To test the modulus, nano-indentation tests were conducted with an MFP-3D-BIO AFM (Asylum/Oxford; Santa Barbara, CA) interfaced to an inverted optical microscope (Axio-Observer Z1, Zeiss). The AFM employed a low noise Z-sensor coupled with an ultra-quiet Z-drive to produce noise in the tip-sample distance <30 pm at 1 kHz bandwidth. To minimize drift and reduce acoustic noise, the inverted optical microscope was mounted on an optical air table with active piezoelectric vibration control (Stacis, TMC, Peabody, MA), housed in an acoustically isolated, NC-25 (Noise criterion) rated room in which the temperature was stabilized to less than ± 0.1 °C over 24 h through radiative cooling. The Z-piezo sensor (Z-sensor) was calibrated using a standard calibration grating (NT-MDT, Moscow, Russia). The deflection sensitivity was calibrated by pressing the tip against a freshly cleaved mica surface and correlating the cantilever deflection to the Z-sensor reading. With these precautions, detector noise is <0.1 nm/√Hz for frequencies above 0.1 Hz.

An AFM cantilever was used to deform a small volume in a voxel. The voxels were created on a #1 cover-glass both with and without cells, immersed within an area defined by a silicone isolator (Grace Bio-Labs) filled with PBS at 37 °C. Typically, voxels were formed with a volume of 50×30×30 μm^3^. Prior to polymerization, the cover glass was solvent-cleaned with acetone, isopropyl alcohol and 18 MΩ de-ionized water, and then conditioned with a 20% oxygen plasma at 25 W (Harrick Plasma) for 3 min. Whereas PEGDA voxels require no surface treatment to adhere, the coverslip used to support the Gel-MA voxels was coated with 3-(trimethoxysilyl) propylmethacrylate.

Two types of cantilevers were used to deform the voxels: one with a blunted conical silicon tip of 10 nm radius (Fig. S2a left, upper inset) with a cone angle of about 2α = 56° (PPP-ContSC, Nanosensors); and another with a tip (CP-PNP-Au, NanoandMore) modified by rigidly attaching a gold microsphere with a 2 μm diameter (Fig. S2a left, lower inset). The spring constants, *k*, typically, 130–260 pN/nm for the sharp tip and 17–43 pN/nm for the gold tip, were determined by measuring the thermal noise spectra and fitting the response to a simple harmonic oscillator. The resonant frequency, *f*_*0*_, and quality factor were obtained by measuring the thermal noise spectra of the unloaded AFM cantilever in liquid (PBS) and fitting the response to a simple harmonic oscillator with an added noise floor: i.e.


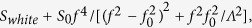
 where Λ represents the quality factor. The cantilever sensitivities were determined consistently to be near 50 nm/V.

The cantilever was positioned directly above a voxel using a 40× telescope and then force-extension curves were measured using a low rate (200–1000 nm/s) *z*-scan at a frequency <1Hz with a range as large as 1 μm, which minimized not only hysteresis but also drag force. Five replicate indentations in the same location were done to establish that the material properties of the voxel did not change with repeated indentations. Finally, the indentation force (*F*) was calculated from Hooke’s law (*F* = *k·d*), where *k* and *d* denote the cantilever’s spring constant and the cantilever’s measured deflection, respectively. The indentation depth (*δ*) was calculated from the difference in the *z-*sensor and the deflection of the cantilever. To minimize mechanical strain of the underlying voxel and to ensure an exclusive indentation of the top of the cell layer only, the maximum applied force was limited to 400–2000 pN.

The indentation force was related to the elastic modulus through the relation: 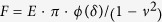 where *E* denotes the apparent elastic modulus and *v* denotes the Poisson ratio^28^. The function 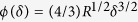 corresponds to a rigid sphere of radius *R* indenting an elastic half-space to a depth *δ*—an adequate model for a microparticle-modified tip used in some of the tests. On the other hand, when an unmodified AFM tip was used, it was represented by a blunt cone with tip angle of 2α, so that:


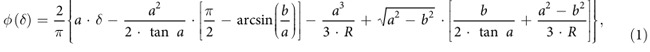


where *a* denotes the radius of contact, *b* is the transition radius, *R* the tip radius and α is half the cone angle of the tip^28^.

### Weighing Hydrogel with an AFM

Voxels without cells were analyzed for swelling ratio, porosity, average molecular weight between crosslinks (*M*_*c*_, g/mol), and mesh size (ξ) by depositing them on an AFM cantilever and weighing them using the change in the resonance frequency. The swelling ratio *Q* and water content were computed from wet weight (*W*_*wet*_) and dry weight (*W*_*dry*_) measurements. Samples were weighed wet, lyophilized for days, and then weighed dry. Two types of cantilevers were used to weigh the voxels: one was a V-shaped, gold-coated nitride probe (BL-TR400PB, Olympus, Japan) with a nominal spring constant of 90 pN/nm (Fig. S2a right, inset), which was used in conjunction with iDrive (Asylum), for easy auto-tuning of the cantilever in fluid; and the other (Fig. S2b right, inset) was a rectangular, silicon single beam cantilever (SSS-FM, Nanosensors) with a nominal spring constant of 2.8 nN/nm.

To weigh the hydrogels, the cantilever was first cleaned in solvents and then the resonance frequency was stabilized after repeated rinses in 18 MΩ de-ionized water. The resonance frequencies, 32.45 kHz and 72.05 kHz for V-shaped and rectangular cantilevers respectively, were each measured 10 times using a sweep width of 10 kHz and drive amplitude of 50 mV and then averaged for the calculation of the loaded mass. Then, a hydrogel was formed on the cantilever (Fig. S2a,b, lower right insets), which resulted in a reduction in the resonance frequency. With the load distributed uniformly along the cantilever, the change in the resonant frequency is proportional to the change in mass (Δ*m*) through the relation: 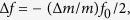
in which the resonance frequency is
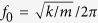
where *m* denotes the mass and *k* the spring constant. The resonant frequency was obtained by measuring the thermal noise spectra of the unloaded AFM cantilever in air and fitting the response to a simple harmonic oscillator. For the V-shaped cantilever, with a resonant frequency of
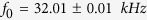
and a mass of 12.4 ng, the mass resolution was about 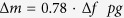. Likewise, for the rectangular cantilever, with 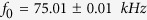 and 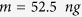, 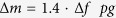.

To accommodate the non-uniform loading of the cantilever associated with the hydrogel the spring constant was modified according to: 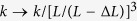, where *L* is the length of the cantilever, and Δ*L* is the distance from the cantilever center-of-mass of the voxel^31^. This accommodation applies for both V-shape and rectangular cantilevers. For the V-shape cantilever weighing PEGDA, *L = *100 μm and Δ*L* = 35 μm, whereas for the rectangular cantilever weighing Gel-MA, *L* = 225 μm and Δ*L* = 60 μm. Thus, the non-uniform load on the cantilever was determined from the frequency shift (Table S1). After the wet weight was measured, the cantilever with a wet equilibrium hydrogel was transferred to the lyophilizer and freeze-dried under conditions of 4 Pa and −87 °C until no further change was observed (4–9 da) and then the dry weight was measured. The swelling ratio 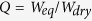, and the porosity (*Q*-1)/*Q* were inferred from these weights.

### Estimating the Mesh Size, ξ

The molecular weight between crosslinks can be approximated using the Peppas-Merrill model according to:^72^


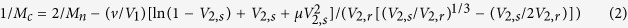


where *M*_*n*_ is the average molecular weight of the polymer, *v* is the specific volume of bulk polymer in the amorphous state, *V*_*1*_ is the molar volume of the solvent, μ is the Flory-Huggins polymer-solvent interaction parameter, and *V*_*2,r*_ and *V*_*2,s*_ are the volume fraction of the polymer at the relaxed state and swelling equilibrium, respectively. *V*_*2,r*_ is equivalent to the volume concentration of the solution where cross-linking occurs and *V*_*2,s*_* = *1/*Q*. Accordingly, the mesh size (ξ) was calculated from: 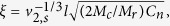
where *l* is the average value of the bond length between C-C and C-O bonds in the polymer repeat unit, *M*_*r*_ is the molecular mass of the repeat unit [-O-CH_2_-CH_2_-], and *C*_*n*_ = 4 is the characteristic ratio for polymer. Similarly, for Gel-MA, Mc was estimated from the Peppas-Merrill model, but with *V*_*2,r*_ = 1. In this case, the mesh size was given by: 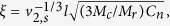
where *l* is the mean of one C-C bond and two C-N bonds (so that the factor 2 was replaced by 3). The parameters characterizing each hydrogel are tabulated in Table S2.

### Microscopic characterization of synthetic capillaries

#### Cross-section of a capillary

A 3D model for each microtubule was obtained from the volume reconstructed from image stacks obtained from a series of confocal images of the cellular array (Imaris, AG Switzerland). Carboxylate-modified, mono-dispersed fluorescent nanospheres (40 nm diameter, FluoSpheres) were perfused through the capillary and attached to cell walls (Molecular Probes, Invitrogen). Generally, the cross-section of the lumen transverse to the capillary axis was irregular, so the area, not the diameter, was measured at 5 μm intervals. The coincidence between these areas and velocities along the capillary axis were obtained for several capillaries, varying the differential pressure across the capillary and average area of the lumen.

#### Velocity and Shear Stress

To flow blood through a capillary, first it was tightly sealed against the pedestals and walls forming the cross-connecting channel in the microfluidic device using 5 kDa PEGDA hydrogel. Then a washed 10% suspension of human erythrocytes in PBS containing sodium citrate was perfused through the capillary using a microfluidic control system (MFCS, Fluigent, Paris, France) to determine the pressure and monitor the flow through it. Focusing on the lumen along the axis of the capillary (*x-y*), time-sequenced data of the trajectories associated with individual erythrocytes was extracted from confocal scanned transmission images. Each frame was subtracted from the previous one to generate a video of the pixels that have changed (Fiji software). After this operation, only the outline of the erythrocytes remained in the image sequence.

These images were subsequently digitally filtered to eliminate noise. The trajectory of each erythrocyte was tracked throughout the capillary by identifying the *x* and *y* coordinates corresponding to the center of the cell in each consecutive frame using the MTrackJ plugin. The axial erythrocyte velocity *V*_*ax*_ was measured using the definition: *V*_*ax*_* = D*_*ax*_*/*Δ*t* where, *D*_*ax*_ denotes the displacement of an erythrocyte between frames, after a short time interval Δ*t,* which corresponds to the frame acquisition rate (8–32 ms). The erythrocyte velocity was averaged and a standard deviation was calculated. For erythrocytes moving slowly, more than 2Δ*t* time increments were used for each *V*_ax_ measurement, so that the minimum distance traveled was about 5 μm. The maximum relative error in the axial velocity measurement can be regarded as the combined effect of the relative error in the measurement of the time interval Δ*t* between two successive images of the flow and the relative error in the measurement of the axial displacement of an erythrocyte, but since the former was negligible (deviations of the frame acquisition rates were less than 0.001%), the total relative error in the axial velocity measurement was equal to the latter, i.e. approximately 5%, which was attributed to the optical resolution of the image (500 nm/pixel). However, whereas the measurement was accurate the actual movements of the RBCs were random as evident from the error bars representing the standard deviation in position.

For erythrocytes flowing in a capillary, there was a net hydrodynamic force pushing the cells towards the center. Erythrocytes did not penetrate the capillary wall, which implies that they must be at least ½ *d*_RBC_ away, where *d*_RBC_ is the diameter of a human erythrocyte. This means that, on average, there will be more erythrocytes near the center of the capillary. The average axial velocity, *V*_*ax*_, and the wall shear rate were measured directly. The average wall shear rate always exceeded 200 s^−1^, which makes the fluid appear Newtonian. In addition, the *in vivo* viscosity law was used for the estimation of *η* to take into account the viscosity change with vessel diameter. The wall shear stress was estimated from Newton’s law, which is just a product of the gradient of the velocity profile with respect to the outward normal *n* of the wall and the kinetic viscosity, *η*, i.e. 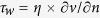, assuming the viscosity of blood for shear rates >100 s^−1^ at 37 °C was given by η = 3.5 mPa·s^56^,^57^. Typically, the shear rate in a capillary ranged up to about 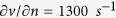.

### Fluorescence Imaging

#### Cells

Fluorescence data was collected using a Leica TCS SP5 II (Leica Microsystems) confocal microscope with an 8 kHz resonant scanner and enhanced, hybrid *GaAsP* (HyD) detectors for improved sensitivity to fluorescence. All confocal images were acquired using either a 63× 1.2 NA or 25× 0.9 NA water immersion objective (Leica) with an argon laser excitation (488 or 514 nm) at 500 nW and diode laser at 564 nm using a pinhole (1 Airy unit). Fluorescent z-stacks (along the optic axis) were recorded. An image matrix comprised 512×512-pixels, each was 0.4 μm thick extending over a 150.0 μm depth. False-color perspective volumes were reconstructed from image stacks obtained from a series of confocal images showing the 3D aspects of the cellular array using Imaris software (Bitplane AG), which rendered fluorescent volumes using an intensity threshold.

The entire confocal microscope including the microscope stage, objective and the microfluidic device, were encased in an environmental chamber (BLX 2000). To maintain the tissue during incubation, the chamber was used to automatically regulate using feedback the temperature (37 ± 0.1 °C) and the partial pressure of CO_2_ (pCO_2_ 5% atm).

#### Immunofluorescence (IF) Labeling of Cells

To visualize cell-specific surface markers, the cells comprising the capillary were stained by direct IF using FITC-conjugated mouse anti-human CD31 monoclonal antibody (303103, Biolegend, San Diego, CA) and CF680-conjugated mouse anti-human chondroitin sulfate monoclonal antibody (554275, BD Bioscience, San Jose, CA). Prior to direct IF, the anti-chondroitin sulfate antibody was conjugated with CF 680 (Biotium, Inc., Hayward, CA). To validate cell-cell adhesion in confluent hMVECs maintained under standard culture conditions, FITC-conjugated mouse anti-human VE-cadherin monoclonal antibody (NBP1-58370, Novus Biologicals, Littleton, CO) was used. For live cell staining, these antibodies were diluted using culture medium and used at the final concentration of 1-2 μg/ml. After incubation at 37 °C, in 5% CO_2_ for 30 min., unbound free antibody was washed with excess culture medium. Attempts were made to label hNFs with CD-90 (Thy1) without marking the pericytes or hMVECs, but contrary to expectation, Thy1 failed to stain hNFs.

#### Oxygen

To rigorously establish the boundary conditions and eliminate spurious oxygen, the microfluidic device was housed in an environmental chamber (Pecon GmbH, Erbach, Germany) that was purged with nitrogen for >1 h for a low oxygen condition or backfilled with oxygen for high level conditions. To preclude out-diffusion of oxygen, the connections to the microfluidic were made with Polyetheretherketone (PEEK) tubing. After a capillary was formed, oxygenated fluid was forced through it using a differential pressure across the inlets; the right-hand port on the inlet of the microfluidic (Fig. 1a) was connected to a high-level oxygenated medium, pO_2_ = 20 kPa, flowing at a 30 μL/min rate, while low-level oxygenated medium, pO_2_ ≤ 2 kPa, flowed at a 0.5 μL/min rate into the left-hand inlet.

The pressure within the microfluidic device was estimated from a finite element analysis using COMSOL. The Navier-Stokes equation was solved, taking into account the tubing from an ELVE control system (Elveflow, Paris, France) to the microfluidic and from the microfluidic to the waste containers and the applied flow rates. It revealed an average flow rate of 0.25 and 0.05 μL/min, corresponding to differential pressures of 1.2 Pa and 0.045 Pa across the capillaries in Fig. 5a,b respectively. The low-level oxygenated solution was prepared by pushing degassed medium purged with dry nitrogen gas through Tygon tubing (ID 500 μm) 1 m long at a flow rate of 50 μl/min, whereas the high-level solution was prepared by pushing medium with atmospheric oxygen through the same Tygon tubing 1 m long at the same flow rate. The solutions were subsequently forced through PEEK tubing to prevent further out-diffusion.

To detect oxygen, a ruthenium dye RTDP (tris(2,2-bipyridyl)dichloro-ruthenium(II) hexahydrate) (0.3% w/v) was used in conjunction with the NeoFOX phase measurement system (Ocean Optics). The excitation light of the pulsed blue LED and a red reference LED with a repetition rate of 94 kHz were focused with a 100× objective onto a ~20 μm spot on the sample and on the lumen of the capillary at a height of 30 μm above the cover slip. The spatial resolution of the measurement was affected by both the spot size and the step-size. The actual step-size represented a compromise between the scan time across 2000 pixels at 700 ms/pixel and the size of the multi-core optical fiber illuminating each pixel.

When excited by the pulsing blue LED, the ruthenium dye transfered energy by collisional quenching to the oxygen and the lifetime was reduced. With an appropriate calibration, the fluorescent lifetime measured by NeoFox can be related to the oxygen concentration using the Stern-Vollmer relationship. The quenching coefficient, 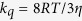, depends on the viscosity η, which differed in hydrogel, tissue and medium. To account for the viscosity in the oxygen profile, two additional calibration scans of the fluorescent lifetimes were acquired: one for which the microfluidic was immersed in a <1% oxygen solution and equilibrated for 12 h; and another for which the microfluidic was immersed in ambient oxygen for 12 h. Temperature also affected the solubility of oxygen in samples, producing a change in the calibration slope. To avoid changes in temperature, the experiments were all conducted at 37.0 ± 0.1 °C.

The sample was scanned with a 10 μm step-size and lifetimes were measured for 700 ms at each step. For a wide field, an area spanning 1240 × 620 μm^2^ was scanned yielding 1922 oxygen pixels, but typically data was acquired from smaller areas along and transverse to the capillary axis. At each point in the scan, a transmission image was captured as well. Finally, all transmission images were stitched together to form a transmission image and overlaid with the corresponding profile.

### Finite Element Simulation of Oxygen transport

COMSOL Multiphysics 4.3 was used to perform finite element simulations of oxygen transport. The diffusion of oxygen was assumed to follow Fick’s laws and so, along with advection, the oxygen concentration throughout the capillary was modeled deterministically according to the equation:


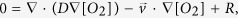
 where [*O*_*2*_] is the local oxygen concentration and *R* denotes an oxygen sink (tissue), the velocity *v* was determined by another simulation using Navier-Stokes equation and incompressible flow with no-slip boundary conditions. The model consisted of a cylindrical tube with a lumen matching the average diameter along the capillary—ranging from 10 to 20 μm—situated 25 μm above the cover glass positioned in the center of a block 50 × 50 μm^2^, 200 μm long. The dimensions of the tube were taken from transmission images focused at the lumen acquired shortly before the oxygen measurements. The wall of the tube represented the hMVECs and hPPs. Another concentric tube 50 μm tall and 75 μm wide and as long as the capillary represented the hNFs. The remaining space between the walls of the microfluidic and cover-glass were filled with hydrogel except at the inlet and outlet where there was blood or medium. The diffusivity of dissolved oxygen, *D*, in the hydrogel polymer, media, and blood was set to 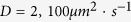, based on a comparison of time-dependent measurements of the oxygen profile within those materials and water—no significant difference was observed^73^. In bulk tissue, the diffusivity was set to 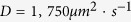. For volumes containing hMVECs, hPPs and hNF, it was assumed that the cells acted as sinks consuming oxygen at a rate of 0.05 mol·m^−3^·s^−1^ in medium containing 0.5 mol·m^−3^. The boundary conditions were chosen to match the measurements. Accordingly, the inlet oxygen was set to 6 and 14 kPa partial oxygen, and the oxygen 100 μm on the *trans*-side of the capillary was set to 1 and 7 kPa and the surfaces in contact with PDMS were set to 2 and 9 kPa, respectively for the capillaries of Fig. 5a,b. The glass bottom of the microfluidic devices was assumed to preclude any oxygen diffusion. For modeling, the 3D geometry of the capillaries were drawn and filled with pre-defined high-resolution grids.

## Additional Information

**How to cite this article**: Sarveswaran, K. *et al.* Synthetic Capillaries to Control Microscopic Blood Flow. *Sci. Rep.* **6**, 21885; doi: 10.1038/srep21885 (2016).

## Supplementary Material

Supplementary Information

Supplementary VIDEO V1

Supplementary VIDEO V2

Supplementary VIDEO V3

Supplementary VIDEO V4

## Figures and Tables

**Table 1:** Hydrogel mechanics.

*Hydrogel*	*Mn*(kDa)	%(w/v)	*Q*(*Weq/Wdry*)	Porosity (*%*)	*Mc*(g/mol)†	ξ (nm)†	*Enocell*(*kPa*)‡ 10 nm/1μm	*Ecell*(*kPa*)‡ 10 nm/1μm
PEGDA5.0k	5.0	10	10.1 ± 1.7	90.1 ± 1.5	900±200	4.5 ± 0.8	105 ± 2/3.7 ± 1.9	112±19/4.0±0.9
Gel-MA*	87.5	8	10.8 ± 1.4	90.7 ± 1.4	18000±4000	21.2 ± 3.3	11.2 ± 1.5/2.5 ± 1.3	35 ± 11/2.0±0.6

The effect of molecular weight (*M_n_*) of PEGDA and Gel-MA hydrogels at the indicated percentage mixture %(w/v), on the swelling ratio, *Q*, the porosity, (*Q-1*)/*Q*, the average molecular weight between crosslinks (*M_c_*), mesh size (ξ) and Young’s moduli with and without encapsulated hMVECs (PEGDA) and hNF (Gel-MA) is shown.

^†^The structural properties of the hydrogels were analyzed using the Peppas-Merrill model.

^‡^These results were obtained by using an AFM with sharp 10 nm radius tip and Au microsphere tips with a 1–3 μm diameter

^*^Gelatin from porcine skin (G1890)has an average molecular weight of 87.5 kDa with a bloom number of 300.
