# Supplementary material for: Synthetic Capillaries to Control Microscopic Blood Flow
Source: Sci Rep. 2016 Feb 24;6:21885. doi: 10.1038/srep21885 (PMC4764836; doi:10.1038/srep21885)
Supplement: Supplementary Information [file srep21885-s1.pdf]

SUPPLEMENTAL MATERIALS

Title: Synthetic Capillaries to Control Microscopic Blood Flow

Authors: Koshala Sarveswaran,† Volker Kurz,† Zhuxin Dong, Steven Penny, Tetsuya Tanaka, Gregory Timp,

Affiliation: Depts. Biological Science and Electrical Engineering, 316 Stinson-Remick Hall, University of Notre Dame, Notre Dame, IN 46556, tel: 574-631-1272, email: gtimp@nd.edu

Hydrogel mechanics measured by nanoindentation and pictogram mass determination.

Infinitesimal strain theory relates the indentation force to the elastic modulus through the relation: $F = E \cdot \pi \cdot \phi(\delta)/(1-v^2)$, where E denotes the elastic modulus, v the Poisson ratio, and $\phi(\delta)$ is a function of the indenter geometry (Methods).[28] However, rigorous application of infinitesimal theory demands an infinitely large specimen relative to both the indentation depth and the radius of the indenter, otherwise nonlinearities arise.

To gauge the stiffness, the elastic modulus (E) was measured by nano-indentation experiments performed with an atomic force microscope (AFM) the indentation depth (δ) was calculated from the difference in the *z*-sensor and the deflection of the cantilever (Fig. S1). Two different probes were used: a blunted conical silicon tip of 7-10 nm radius (designated as the "sharp tip," Fig. S2a left, upper inset); and a tip modified by rigidly attaching a gold microsphere with a 1.5-3 μm diameter (designated at the "gold tip," Fig. S2a left, lower inset). To measure the elastic modulus, voxels with and without cells in them were positioned under the cantilever, and then a small volume was deformed by extending the cantilever while the deflection was measured. The resulting indentation force (F) was calculated from Hooke's law.

Regardless of the tip and the voxel size and shape, the data consistently showed a linear relationship between the indentation force and the function ϕ (Figs. S2a-c, left). However, the sharp tip consistently showed a much higher modulus compared to the gold tip (Table Interestingly, the mechanical properties of voxels incorporating cells can reflect either the cell or the gel modulus, depending on the scale of the measurement (Table 1). The apparent elastic modulus for a voxel including an hMVEC was determined to be $E_{PEG+cell} = 110 \pm 20$ kPa (Fig. S2c, left) using a sharp tip, which seems to reflect the PEGDA modulus described above, not that of an EC. In contrast, indentation with a gold tip revealed a modulus of $E_{PEG+cell} = 4.0 \pm 0.9$ kPa, similar to other ECs measured alone[24] or just PEGDA measured with a gold tip. In contrast, the modulus of a Gel-MA voxel incorporating an hNF measured with a sharp tip was $E_{Gel+cell} = 35 \pm 1$ kPa, which was similar to that of a fibroblast,[25] whereas the stiffness measured with a gold tip was only 2.0 ± 0.6 kPa, which was more indicative of Gel-MA.

A lower modulus implies a sparser cross-link density in the gel and higher diffusivity through it. To corroborate the modulus measurements, the mesh size was inferred from the swelling ratio $Q = W_{eq}/W_{dry}$ obtained by weighing a voxel to determine equilibrium weight (W_{eq}) and dry weights (W_{dry}).[31] To achieve sub-picogram resolution, the weight was inferred from the shift in the resonant frequency of an AFM cantilever that occurs when the cantilever was loaded with a voxel. When cantilevers were loaded with PEGDA or Gel-MA, a shift in the resonant frequencies of Δf_{dryPEG} = -0.29±0.02 kHz, Δf_{wetPEG} = -2.60±0.05 kHz and Δf_{dryPEG} = -0.29±0.02 kHz, and Δf_{dryGEL} = -6.2±0.1 Hz and Δf_{wetGEL} = -67±9 Hz were observed (Figs. 2a,b, right). For an accurate mass estimate, the shifts were corrected to account for non-uniform distribution of the load (Table S1). Subsequently, the shifts were translated to estimates for Q: i.e. Q_{PEG} = 10.12 and Q_{GEL} = 10.75, respectively. The molecular weight between cross-links (M_C) was determined from the swelling ratio using the Peppas-Merrill model (see Methods), which was then used to estimate the mesh size (Tables 1, S2). It was observed that ξ was statistically larger for Gel-MA, $\xi = 21.2\,nm$ compared to PEGDA $\xi = 4.5\,nm$, implying a sparser cross-link density, which corroborates the lower modulus observed in the nano-indentation experiments.

The corresponding porosity, $(Q-1)/Q$, which was estimated to be 90.1% and 90.7% for PEGDA and Gel-MA respectively, factors into the effective diffusion coefficient through the porous medium. To test this last assertion, the diffusion of a surrogate protein, fluorescent streptavidin, was tracked through the hydrogels using confocal microscopy (see supplement and Figs. S3a,b). Growth factors secreted from hMVECs or hNFs, such as VEGF and bFGF, have molecular weights of 38.2 kDa and 17.4 kDa respectively, whereas the average molecular weight of secreted protein in the human secretome is 41.9 kDa so that streptavidin with a molecular weight of 68 kDa, with a corresponding volume of 133 nm^3, makes a suitable surrogate. The measured values for the diffusivities of $D_{str}^{PEG} = 1.6 \pm 0.1\,\mu m^2$ and $D_{str}^{Gel-MA} = 31 \pm 5\,\mu m^2$ for PEGDA and Gel-MA respectively, were diminished from the free diffusion of the same ligand in water,[42] i.e. streptavidin diffusivity in PEGDA was less than 2% of the bulk value, $D_{str}^{water} = 130 \pm 10\,\mu m^2$, which indicates a mesh size comparable to the size of the solute. Thus, a larger mesh size implies a higher diffusivity for nutrients and waste. However, due to the inhomogeneous distribution of water in a voxel containing a cell, it may be necessary to invoke more general kinetics beyond Fick's law to precisely account for material transport.

FIGURE S1.

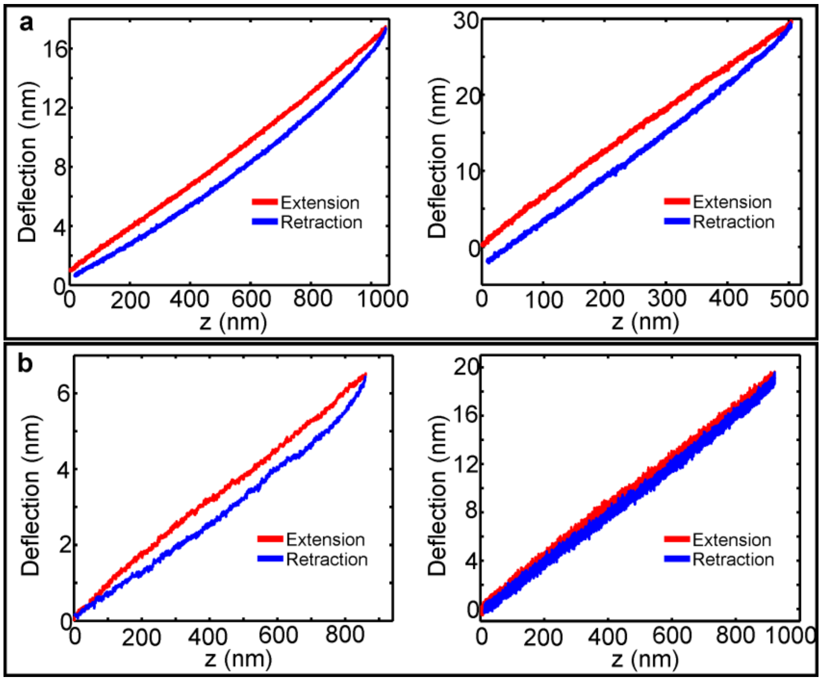

FIGURE S1. Nanoindentation deflection versus z-height (depth) measured in (a) hMVECS and (b) hNF voxels. (a, left) Representative plot of the deformation measured using a sharp AFM tip. **(a right)** Like (a, left), but using a tip modified with a gold microsphere. **(b, left)** Representative plot of the deformation measured using a sharp AFM tip. **(b right)** Like (b, left), but using a tip modified with a gold microsphere.

FIGURE S2.

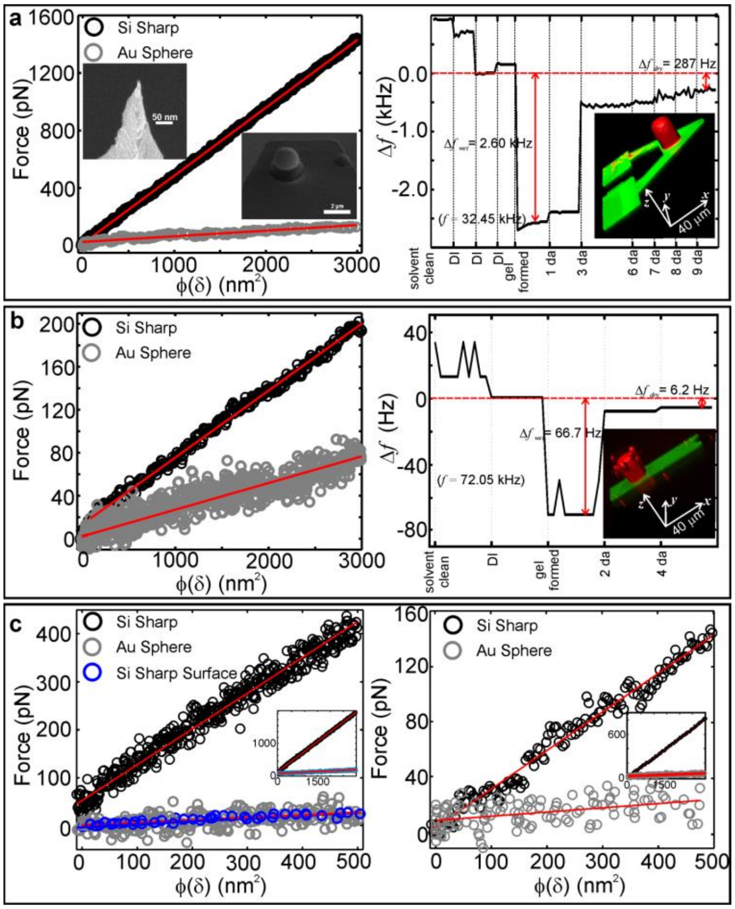

FIGURE 2. Nano-indentation force versus the tip function $\phi(\delta)$ defined in the text, and picogram mass deviation measured with an AFM in a PEGDA and Gel-MA voxels with and without cells. (a,left) Representative plot of the linear regression fit of the deformation force measured with both sharp silicon and micro-spherical gold tip in a PEGDA voxel as a function of ϕ. The force-indentation data fit classical infinitesimal strain theory (red solid lines). The moduli were inferred from the slopes of the fits. **(a,left) Inset top:** SEM image of the blunted conical tip showing the indenter radius (10 nm) with a tip angle $2\alpha = 56°$. **(a,left inset bottom)** SEM image of the microspherical gold indenter with a 2 μm diameter. **(a,right)** Dependence of the resonance frequency of a BL-TR400PB cantilever following a solvent clean (1), after loading with a (wet) 5.0 kDa PEGDA hydrogel (2), and drying in a lyophilizer (3). The frequency shift corresponds to a change in the mass on the tip. **(a, right inset)** Reconstruction obtained from a confocal image of a PEGDA hydrogel stained by fluorescent rhodamine attached to a BL-TR400PB cantilever. **(b,left)** Like (a,left), but for the deformation of Gel-MA voxel instead. The solid lines represent linear regression fits of the force as a function of the coefficient ϕ to the classical infinitesimal strain theory. The moduli were inferred from the slopes of the fits. **(b,right)** Like (a,right), but weighing a Gel-MA hydrogel instead using an SSS-FM

cantilever. **(b, right inset)** 3D confocal microscope image of a Gel-MA hydrogel stained by fluorescent rhodamine attached to a SSS-FM cantilever **(c,left)** Like (a,left), but for the deformation of voxel consisting of PEGDA encapsulating an hMVEC instead. The moduli were inferred from the slopes of the fits. **(c, left inset)** The same data plotted over a larger indentation range. **(c,right)** Like (c,left), but for the deformation of voxel consisting of Gel-MA encapsulating a fibroblast. The moduli were inferred from the slopes of the fits. **(c, right inset)** The same data plotted over a larger indentation range.

Table S1. The shifts in resonant frequency Δf of the cantilever for dry and wet hydrogels, and the corresponding wet and dry masses, *m*. For convenience, the calculated swelling ratio, *Q*, and porosity, (*Q*-*1*)/*Q* are repeated.

Hydrogel	M_n(kDa)	Δf_{dry}(Hz)	Δf_{wet} (Hz)	m_{dry} (pg)†	m_{wet} (pg)†	$Q(W_{eq}/W_{dry})$	Porosity (%)
PEGDA$_{5.0k}$	5.0	287±24	2600±48	10.7±1.3	108.1±2.7	10.12±1.67	90.12±1.47
Gel-MA*	87.5	6.2±0.1	66.7±8.8	0.93 ±0.12	9.99±1.31	10.75±1.42	90.69±1.41

† These results were obtained using two types of cantilevers. To weigh the PEGDA a V-shaped, gold-coated nitride probe (BL-TR400PB) with a nominal spring constant of 90 pN/nm was used, whereas a rectangular, silicon single beam cantilever (SSS-FM) with a nominal spring constant of 2.8 nN/nm was used to weigh the Gel-MA.

*** Gelatin from porcine skin (G1890) has an average molecular weight of 87.5 kDa with a bloom number of 300.**

Table S2. The parameters used to calculate the molecular weight between cross-links (M_n) and mesh size (ξ) for PEGDA and Gel-MA. The parameter *l* represents the average value of the bond length between C-C and C-O bonds in the polymer repeat unit; M_r is the molecular mass of the repeat unit; C_n is the characteristic ratio for polymer, *v*, is the specific volume of bulk polymer in the amorphous state, V_1 is the molar volume of the solvent, $V_{2,r}$ and $V_{2,s}$ are the volume fraction of the polymer at the relaxed state and swelling equilibrium, and μ is the Flory-Huggins parameter, respectively.

Hydrogel	*l* (*nm*)	M_r (*g/mol*)	C_n	*v* (*cm*^{3}/*g*)	V_1 (*g/mol*)	V_{2s} (=Q)	V_{2r} (%)†	μ (nm)
PEGDA$_{5.0k}$	0.146	44	4.0	0.893	18	0.099	14.3	0.426
Gel-MA$_{87.5k}$	0.139	94.7	8.26	0.741	18	0.093	100	0.497

†$V_{2,r}$ is equivalent to the volume concentration of the solution where cross-linking occurs. It represents the gel immediately following polymerization, but prior to the introduction of the gel to a solvent.

Mass uptake of streptavidin in 5.0 kDa PEGDA and Gel-MA.

Mass uptake measurements of a fluorescent proxy (streptavidin, Alexa Fluor 532 conjugate, Life Technologyies) with a similar molecule weight (M.W. 58 kDa) was accomplish in 5.0 kDa PEGDA and 87.5 kDa Gel-Ma hydrogel microstructures of approximately the same size (i.e. 30μm×30μm×30μm) formed under the same conditions used in the experiments in the manuscript. The microfluidic was imaged in a Leica TCS SP5 II (Leica Microsystems) confocal microscope to measure the diffusion of streptavidin into the hydrogels. Streptavidin was injected

at 1 μL/min using a syringe pump. To measure the uptake of streptavidin into the gel, a 100 μm-long line scan along the x-axis was continuously acquired using a 63× water immersion objective excited with an argon ion laser. The mean intensity of the line segment within the hydrogel is plotted versus time (blue line) (Figs. S3a,b). Once the data was acquired, it was fit (red line) using custom MATLAB code using a model for the mass transport in a porous sphere with a diameter *a* and diffusivity *D*, the ratio of the mass uptake, *M*(*t*), relative to the mass at infinite time *M*(∞):

$$\frac{M(t)}{M(\infty)} = 1 - \frac{6}{\pi^2} * \sum_{n=1}^{\infty} \frac{1}{n^2} \exp(-Dn^2\pi^2 t / a^2),$$

This procedure was used to place bounds on the diffusivity in the hydrogel to values in the range: i.e. $D^{PEG}_{streptavidn} = 1.6 \pm 0.5\ \mu m^2/s$ and $D^{Gel\text{-}MA}_{streptavidn} = 30.9 \pm 5.0\ \mu m^2/s$ for 5.0 kDa PEGDA and Gel-MA, respectively. For comparison, the red lines in Fig. S3 represent fits to the data using these diffusion coefficients.

FIGURE S3.

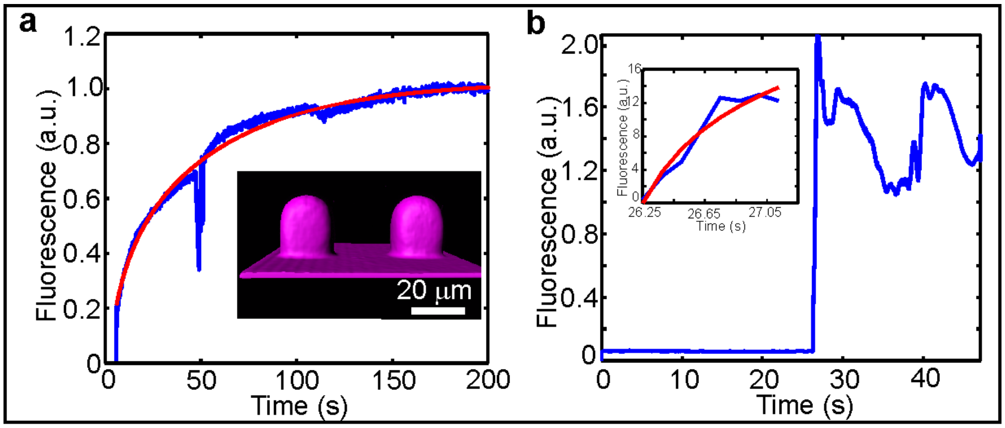

FIGURE S3. Uptake of fluorescent streptavidin in hydrogel microstructures. (a) Typical mass uptake (blue line) of a fluorescent proxy (streptavidin) in 5.0 kDa PEGDA. Fit to the uptake (red line) restricts the range of the diffusion coefficient to: $D^{PEG}_{streptavidn} = 1.6 \pm 0.5\ \mu m^2/s$. **Inset:** Image of the fluorescence observed in a 30 μm-scale hydrogel microstructure after 90 s following a flush with PBS. **(b)** Typical mass uptake (blue line) of a fluorescent proxy (streptavidin) in Gel-MA. Fit to the uptake (red line) restricts the diffusion coefficient to: $D^{Gel\text{-}MA}_{streptavidn} = 30.9 \pm 5.0\ \mu m^2/s$. **Inset:** Magnified view of the uptake near 26 s and the corresponding fit to the data.

FIGURE S4

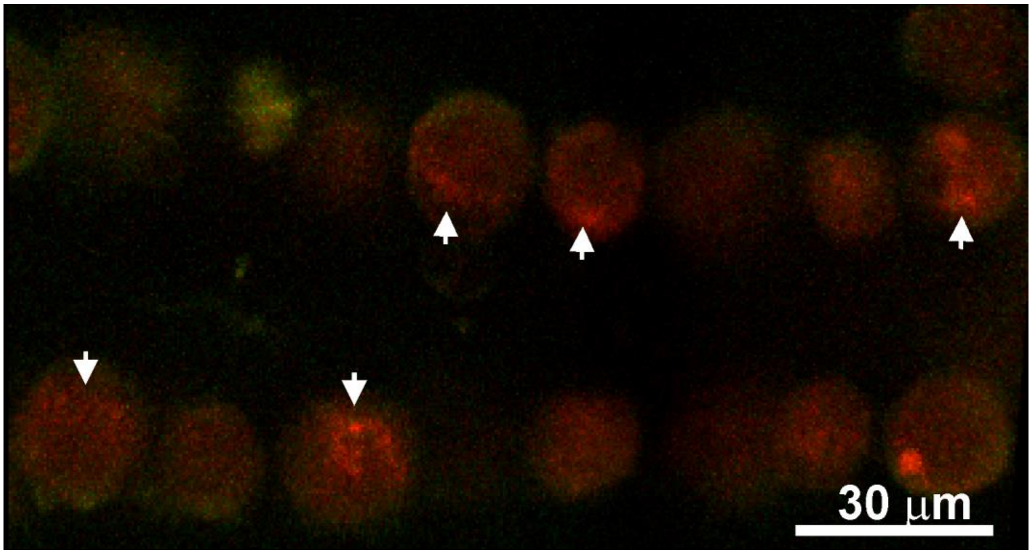

FIGURE S4. Intracellular distribution of Golgi body in the middle layer of a synthetic capillary shown by BODIPY-FL-C5-ceramide staining. The Golgi apparatus is localized near the lumen due to transmural pressure, while the nucleus is forced away from the lumen**.** Sections 1 μm thick were taken along the z-axis by confocal microscopy. Notice that because only 3 consecutive z-slices were used to generate this image, not all of the hMVECs have the localization of Golgi apparatus detected.

FIGURE S5.

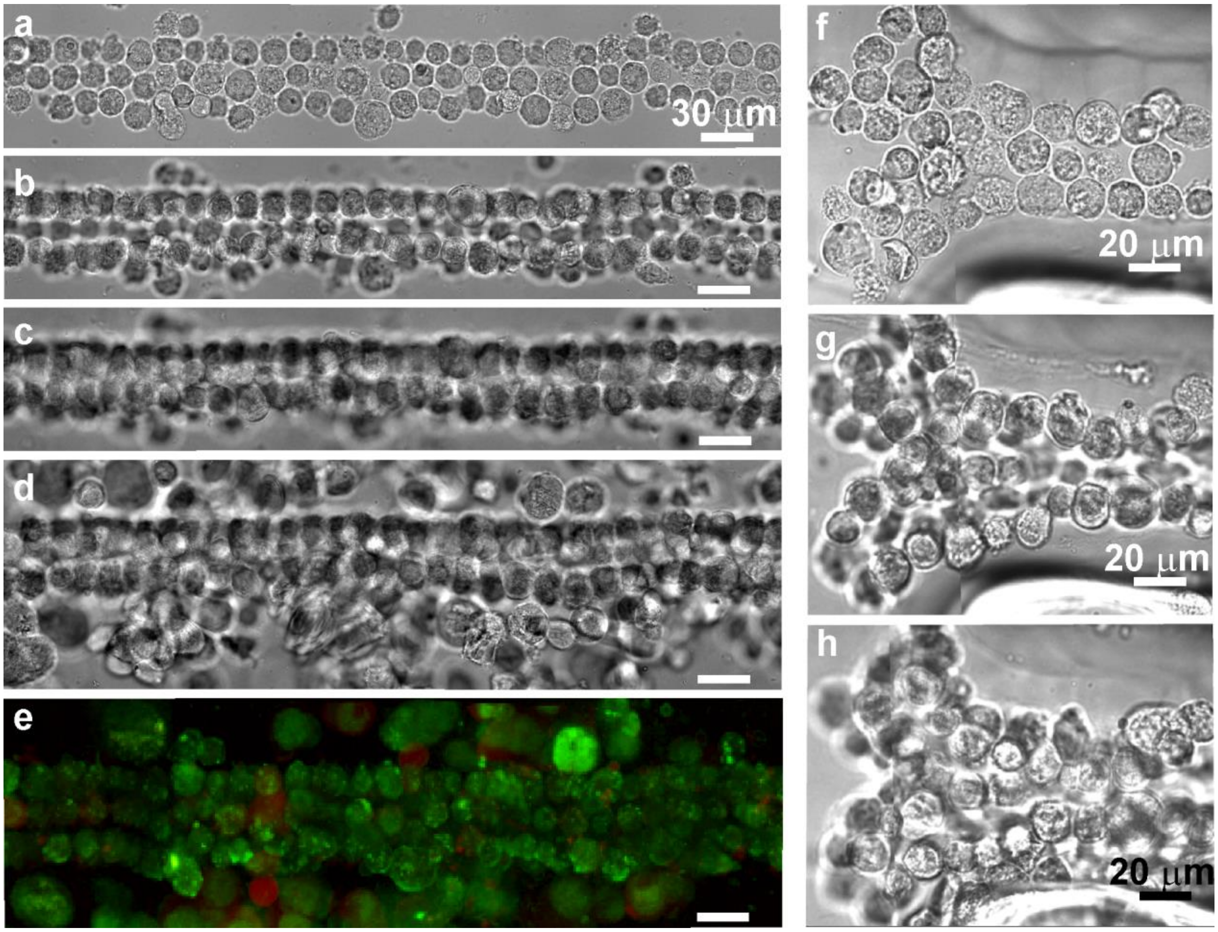

FIGURE S5. Layer-by-layer assembly of half-millimeter long and branched synthetic capillary. (a-c) Top-down transmission optical micrographs of a 3-layer structure of hMVECs partially encapsulated in PEGDA hydrogel showing the basement layer, the lumen and the attic layer respectively, organized using optical tweezers to form a capillary nearly half-millimeter long. **(d,e)**Top-down transmission micrograph and fluorescence micrograph of the same capillary as above after hPPs encapulated in PEGDA-PEG-RGDS and hNFs encapsulated in Gel-MA were added. **(f-h)** Like (a-c), top-down micrographs of a 3-layer structure of hMVECs partially encapsulated in PEGDA hydrogel showing the basement layer, the lumen and the attic layer respectively, organized using optical tweezers to form a branched capillary.

FIGURE S6

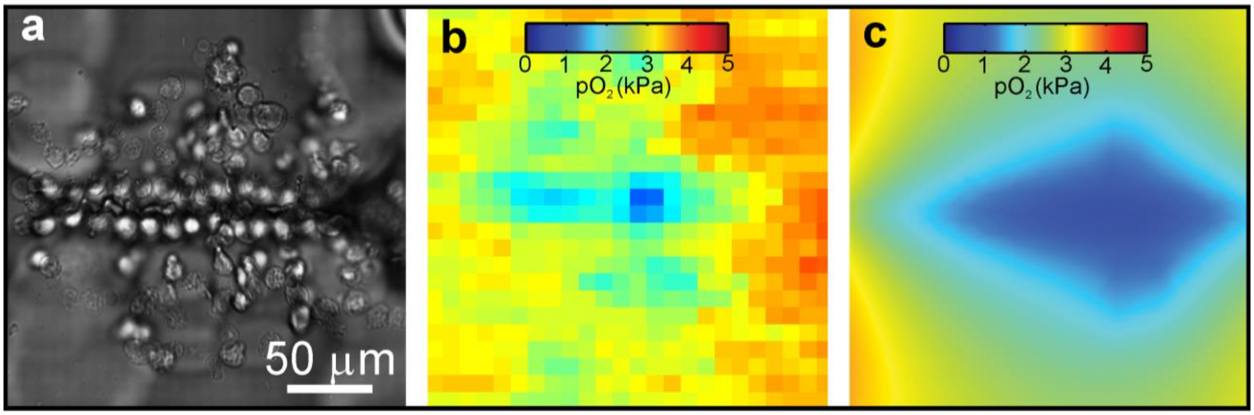

FIGURE S6. Bulk living tissue consuming oxygen while surrounded by high oxygen media without flow through the capillary. **(a)** Transmission image of synthetic tissue assembled from hMVECs and hNFs using the same technique as for capillaries. **(b)** Similar to (a), but a reconstructed heat map delineating the oxygen concentration as measured while oxygenated liquid was flowing over and around the microstructure. **(c)** FES of a microstructure resembling (a) taking into account, diffusion, migration, consumption of oxygen molecules by the cells.

FIGURE S7.

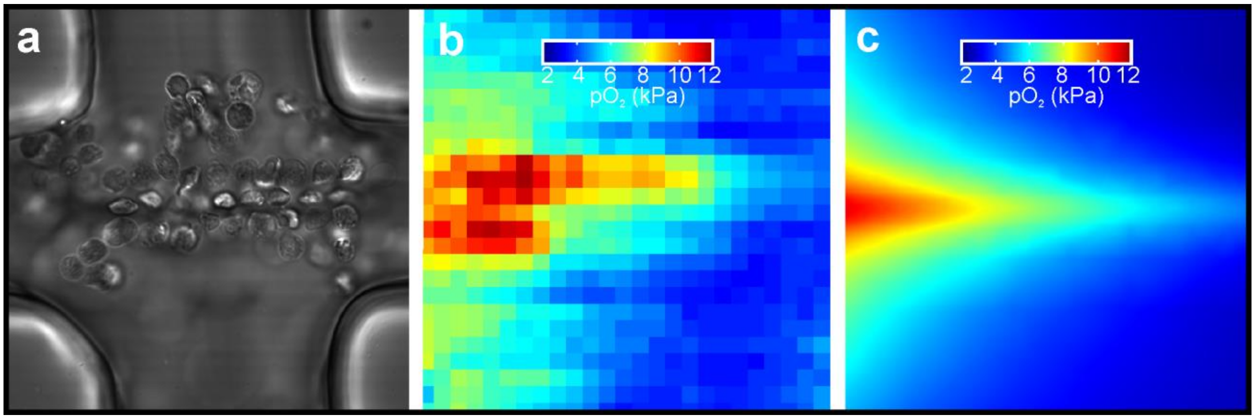

FIGURE S7. Oxygen flow through and gradients across a synthetic capillary. (left) Tiled transmission image of a synthetic capillary embedded in a microfluidic device. The lumen was assembled from HMVECs and hPPs, whereas the abluminal cells were fibroblasts. Rectangular PDMS supports (corners) were used for mechanical support. The capillary has a lumen with a 181 μm^2 cross-sectional area and experienced a flow of 0.10 µl/min. **(b)** Similar to (a), but a reconstructed 2D heat map of the oxygen profile acquired from the structure on the left. The measurements of the oxygen concentration were accomplished in 10 µm steps. **(c)** FESs of the oxygen flow through an idealized capillary under flow conditions similar to that in (a) taking into account, diffusion, migration, consumption of oxygen molecules by the cells.

VIDEOS V1, V2. Washed 10% human erythrocytes (red blood cells) perfusing through synthetic capillaries 180 μm^2 and 560 μm^2 in cross-sectional area, respectively. The erythrocytes flow through the capillary under pressure of 1 kPa. The images were acquired every 20 ms. The mean axial velocity of the erythrocytes under these conditions was about V_{ax} = 0.90 mm/s (V1) and V_{ax} = 0.082 mm/s (V2).

VIDEO V3. Time-lapse imaging of a metastatic breast cancer cell (MDA-MB-231) in a synthetic capillary. Images were acquired every 10 ms using a confocal microscope, although specific z-height was chosen. The video shows initial 4 hr after imaging was started.

VIDEO V4. Human whole blood perfusing through a synthetic capillary. Whole human blood perfusing through a synthetic capillary about 310 μm^2 in cross-sectional area. The blood flowed through the capillary under a differential pressure of 2.5 kPa. The video was compiled from a series of confocal frames through the lumen of the capillary acquired every 16 ms; the total duration was 150 s.
